# A demo‐genetic model shows how silviculture reduces natural density‐dependent selection in tree populations

**DOI:** 10.1111/eva.13606

**Published:** 2023-10-13

**Authors:** Claire Godineau, Victor Fririon, Nicolas Beudez, François de Coligny, François Courbet, Gauthier Ligot, Sylvie Oddou‐Muratorio, Leopoldo Sanchez, François Lefèvre

**Affiliations:** ^1^ URFM, INRAE Avignon France; ^2^ AMAP, Univ Montpellier, CIRAD, CNRS, INRAE, IRD Montpellier France; ^3^ ULiège, Gembloux Agro‐Bio Tech Gembloux Belgium; ^4^ ECOBIOP, INRAE Saint Pée sur Nivelle France; ^5^ BIOFORA, INRAE Orléans France

**Keywords:** demo‐genetic agent‐based model, eco‐evolutionary feedback, evolutionary rate, forest, microevolution

## Abstract

Biological production systems and conservation programs benefit from and should care for evolutionary processes. Developing evolution‐oriented strategies requires knowledge of the evolutionary consequences of management across timescales. Here, we used an individual‐based demo‐genetic modelling approach to study the interactions and feedback between tree thinning, genetic evolution, and forest stand dynamics. The model combines processes that jointly drive survival and mating success—tree growth, competition and regeneration—with genetic variation of quantitative traits related to these processes. In various management and disturbance scenarios, the evolutionary rates predicted by the coupled demo‐genetic model for a growth‐related trait, vigor, fit within the range of empirical estimates found in the literature for wild plant and animal populations. We used this model to simulate non‐selective silviculture and disturbance scenarios over four generations of trees. We characterized and quantified the effect of thinning frequencies and intensities and length of the management cycle on viability selection driven by competition and fecundity selection. The thinning regimes had a drastic long‐term effect on the evolutionary rate of vigor over generations, potentially reaching 84% reduction, depending on management intensity, cycle length and disturbance regime. The reduction of genetic variance by viability selection within each generation was driven by changes in genotypic frequencies rather than by gene diversity, resulting in low‐long‐term erosion of the variance across generations, despite short‐term fluctuations within generations. The comparison among silviculture and disturbance scenarios was qualitatively robust to assumptions on the genetic architecture of the trait. Thus, the evolutionary consequences of management result from the interference between human interventions and natural evolutionary processes. Non‐selective thinning, as considered here, reduces the intensity of natural selection, while selective thinning (on tree size or other criteria) might reduce or reinforce it depending on the forester's tree choice and thinning intensity.

## 1 INTRODUCTION

Eco‐evolutionary feedback loops, which often interact with human interventions, receive increasing attention in the current context of rapid environmental changes (Hendry, 2017). In natural and artificial populations managed over multiple generations, human interventions on populations' composition, structure and dynamics have multiple impacts on evolutionary drivers, i.e., genetic drift, selection and gene flow. Interactions between management and eco‐evolutionary processes mainly proceed from three mechanisms, which can act in concert. First, evolutionary consequences result from the demographic impacts of management on population size, such as when demographic reinforcement in conservation programs avoids an extinction vortex (Seddon et al., 2014), or, conversely, when reduction of effective population size in harvested populations leads to genetic erosion (Allendorf et al., 2008), or when large‐scale release of translocated individuals changes population composition and structure with possible adverse genetic consequences (Laikre et al., 2010). The second mechanism results from the management of the genetic diversity itself, e.g., genetic rescue in conservation programs (Whiteley et al., 2015), or directional anthropogenic selection pressure in harvested populations (Coltman et al., 2003). The third mechanism results from the impacts of management on populations' structure and dynamics, which may interfere with natural selection processes: this type of evolutionary impact of management has received less attention (but see Bouffet‐Halle et al., 2021). In this work, we present a simulation tool to study these three mechanisms of interactions in forest tree populations, and we use it more specifically to characterize and quantify the third one, i.e., the role of tree thinning as a potential “regulator” of natural density‐dependent selection regardless of any direct anthropogenic selection. Understanding this role is needed to develop a stewardship approach to evolutionary management that benefits from and cares for natural evolutionary processes (Mathevet et al., 2018).

Maintaining the potential for genetic responses is acknowledged as crucial for short‐ and long‐term adaptation because plastic responses may not be enough to keep pace with contemporary rapid environmental changes (Alberto et al., 2013). Yet, predicting short‐ and long‐term evolution in managed populations of long‐lived species like trees is particularly challenging because: (i) longer life cycles offer more opportunities for stochastic disturbance events; (ii) the intensity of evolutionary drivers (e.g., viability or fecundity selection) may change over time within a generation in response to disturbance and management interventions; (iii) the short‐term, within generation selective changes of genetic composition, do not linearly translate into long‐term, among‐generation evolutionary rates; and (iv) there may even be trade‐off between adaptation to current versus future environmental conditions, further challenging management decisions such as the choice of the genetic material for afforestation. However, knowledge of the evolutionary impacts of management, their underlying mechanisms, and, whenever possible, a quantitative assessment of these impacts are urgently needed to elaborate exploratory scenarios covering a wide range of plausible outcomes to guide management decisions in the context of global change (IPBES, 2016). In the case of forest management, Achim et al. (2022) defined silviculture as “the science of observing forest condition and anticipating its development to apply tending [i.e., mostly thinnings] and regeneration treatments adapted to a multiplicity of desired outcomes in rapidly changing realities”. A general framework characterizing the pathways from silviculture interventions to their evolutionary impacts has been proposed (Lefèvre et al., 2014), opening the way towards adaptive forest management strategies with a combined objective to speed up adaptation to environmental change while preserving genetic diversity to cope for an uncertain future. However, a quantitative assessment of those short‐ and long‐term evolutionary impacts of silviculture is lacking.

Demo‐genetic agent‐based models (DG–ABM) are efficient tools to address these challenges. Defined as individual‐based population dynamics models with heritable trait variation and phenotype‐dependent interactions between individuals, DG–ABM proved to be useful for the exploration of fundamental questions in evolutionary ecology, and also as effective prospective and decision support tools to investigate the interactions between management and natural eco‐evolutionary processes (Coulson et al., 2006; Lamarins et al., 2022). Following this approach, we developed a forest dynamics model with heritable trait variation, named Luberon2, to simulate the evolutionary processes in monospecific stands with silviculture and stochastic disturbance. In this model, natural selection is dynamically driven by individual tree growth in relation to survival and mating success. Hence, Luberon2 is typically designed to investigate the genetic evolution of traits related to growth, survival or reproduction over a few generations.

A major challenge in such predictive modeling exercise is the validation of the simulated evolutionary dynamics. Luberon2 combines a forest growth and demographic model with a quantitative genetic model of individual variation and heredity. Growth and demographic processes are individually calibrated on empirical data, and a finite quantitative trait loci (QTL) genetic model is used to reproduce observed within‐stand phenotypic variation and heredity. Although each of these components can be separately validated, assessing the validity of joint predictions when all processes are combined remains challenging for three reasons. First, introducing genetic variation, i.e., non‐stochastic and inherited inter‐individual variation, may affect the predictions of the forest growth and dynamics and progressively deviate from the previous calibration domain after several generations. Second, the coupled model aims at exploring complex eco‐evolutionary feedbacks that may result in non‐monotonic genetic trajectories hardly comparable to empirical data. Third, multiple levels of stochasticity in the demographic and inheritance processes may result in uncertain predictions. Multigeneration demographic and genetic records are available to validate predictions of such coupled models in some managed population systems (e.g., Mathieu‐Bégné et al., 2019; Thomas et al., 2016) but this is not yet the case for long‐lived organisms like trees. However, even if the predictions of demo‐genetic models cannot be compared to multi‐generational empirical data for trees, it remains possible to compare the predicted evolutionary rate with empirical estimates for other organisms. Here, we used the standard measure of evolutionary rate proposed by Gingerich (1993), i.e., the average evolution of a trait per generation in units of phenotypic standard deviations, to compare the evolutionary rate predicted by Luberon2 with reviews of empirical estimates in wild populations (Bone & Farres, 2001; Gingerich, 2009; Hendry et al., 2008).

**FIGURE 1:**
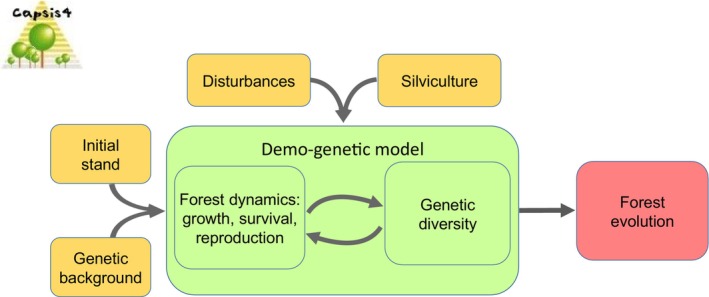
Schematic representation of the eco‐evolutionary feedback loop simulated by the demo‐genetic model (green box): individual tree fitness components, i.e. growth, survival and reproduction capacities, determine forest dynamics and stand structure, which in turn determine the conditions of density‐dependent selection driving the genetic evolution of the population. This dynamic feedback loop is driven by several factors (orange boxes): the initial stand structure, the genetic diversity and genetic architecture of the traits, the disturbance regime and the silvicultural scenario. It results in forest evolution (red box) in terms of demography, performance and genetic diversity.

Here, we investigated an eco‐evolutionary feedback loop (Figure 1) by assessing the rate of evolutionary change of a trait representing the growth component of fitness, vigor. We defined vigor as the deviation of individual diameter increment to the baseline prediction by the tree growth model, which is under quantitative genetic and environmental control. Competition‐driven mortality in managed or unmanaged forest trees, named self‐thinning, is a typical case of density‐dependent soft selection (Bell et al., 2021) where the smallest trees (in relative terms) are eliminated. This direct selection on tree size translates into an indirect selection on our trait of interest, vigor and the correlation between vigor and tree size progressively increases with age. We first characterized the natural process of indirect selection on vigor through competition and mating success in scenarios without silvicultural interventions and considering various quantitative genetic backgrounds. We also quantified the feedback effect of the evolution of vigor on forest productivity and tree size. Then, over four silvicultural cycles corresponding to four generations of trees, we simulated different silvicultural scenarios of random (non‐selective with respect to any heritable trait) thinnings and disturbance regimes. In these simulations, thinning and disturbances were not directly selective and their only potential impact was to reduce the intensity of density‐dependent viability selection and fecundity selection. We assessed the relevance of evolutionary rates on vigor predicted by demo‐genetic coupling with and without silviculture and disturbance in comparison to empirical estimates found in the literature, and we compared the silvicultural scenarios and disturbance regimes with regard to their impacts on evolutionary rates.

## 2 MATERIALS AND METHODS

### 2.1 Purpose and general description of the model

Luberon2, derived with major changes from a previous model Luberon (Dreyfus et al., 2005), simulates the evolution of demographic, dendrometric (various dimensions of trees) and genetic variables at forest scale, over multiple tree generations and under different scenarios. These scenarios are defined by: (i) genetic architecture of variable traits, (ii) spatial environment, (iii) initial population structure and genetic diversity, (iv) disturbance regime and (v) thinning regime.

To that aim, we developed Luberon2 by coupling a forest dynamics model with a model of disturbance regimes and a quantitative genetic model of trait inheritance (Figure 1). Each annual step in Luberon2 corresponds to an eco‐evolutionary feedback loop where: (i) genetic diversity determines the distribution of individual tree performances, i.e., growth, survival and reproduction; (ii) individual performances drive the stand dynamics and structure; (iii) which in turn affect the intensity of the evolutionary drivers shaping the genetic diversity, i.e., genetic drift and selection. The distribution of individual tree performances is also driven by environment, stochastic disturbances and silvicultural interventions, i.e., thinning. Disturbances are modeled through their regimes of impacts, i.e., the frequency and intensity of non‐selective mortality events with a dynamic control by stand‐level and tree‐level characteristics. Luberon2 simulates within‐generation dynamics and inter‐generational evolution of monospecific even‐aged stands, or irregular stands with even‐aged substructure. The variable traits with quantitative genetic inheritance are parameters of forest dynamics processes that ultimately drive individual tree survival and reproduction: vigor is the variable trait in the following simulations. Diverse thinning strategies can be simulated to regulate stand dynamics. The model is developed on the Capsis modeling software platform (Dufour‐Kowalski et al., 2012). Here, we provide a synthetic presentation of the model version calibrated for Atlas cedar, *Cedrus atlantica*, a Mediterranean mountain conifer species (the model gets its name from the emblematic cedar forest of Luberon, Southeast of France), but other species can be considered. Full description of the model is available online in a user manual, regularly updated with new developments (https://capsis.cirad.fr/capsis/help_en/luberon2).

#### 2.1.1 Objects and their variables

The simulated forest stand consists of individual trees and geographic elements (see Appendix S1 for an illustration of the spatial elements described here). Each individual tree belongs to a parcel, which is an area of the spatially homogeneous environment defined by a spatial polygon and an index of environmental quality, here measured by the site index: height of the tallest trees at a given age. The parcel is also a management unit. To allow for spatial heterogeneity in the stand structure and to proceed with regeneration, the stand is split into a regular grid of square pixels. During the simulations, each pixel is modeled as an independent even‐aged stand. Growth, competition and survival processes within a pixel have no impact on neighboring pixels. The only interaction among pixels occurs during the regeneration phase through seed and pollen flow. Pixel size is configurable (15 m × 15 m by default), with possible consequences on the prediction of stand‐scale dendrometric variables (Appendix S2A).

Individual trees have fixed attributes, such as spatial coordinates, multi‐locus genotype, parents' identity, as well as dynamic attributes that evolve during simulations, including age, dendrometric and fecundity variables, the genotypic and phenotypic value of each variable trait (see the user manual for a detailed list of individual tree attributes). The initial individual trees are defined in the inventory input file, and then new individual trees are created by the model during the regeneration process. In the initial inventory, if the individual tree diameters are not provided by the user, they are drawn from the expected distribution of diameters given tree age and stand density.

#### 2.1.2 Succession of processes in a simulation step

Simulations proceed at a 1‐year temporal resolution. Each year, the succession of processes is as follows: growth, selective mortality through self‐thinning, non‐selective mortality through disturbance, regeneration and non‐selective thinning at the end of the annual step (Appendix S1).

#### 2.1.3 Growth and self‐thinning model

In each pixel, the annual diameter growth of each individual tree is computed by correcting the baseline prediction of a distance‐independent tree growth model with an additive term representing the individual phenotypic value of vigor that accounts for genetic and environmental effects.

Here, we used a generic and flexible tree growth model for pure even‐aged stands (Deleuze et al., 2004; Dhôte, 1991), specifically calibrated for Atlas cedar (Courbet, 2002). We simulated a pure even‐aged stand structure not only as a classical management system for this species but also because its evolution is directly comparable to classical quantitative genetics expectations with non‐overlapping generations. Accounting for environmental conditions, tree age and competition, the growth model computes the stand‐level annual increment in basal area, i.e., the cross‐sectional area of trees at 1.3 m above ground. It then converts stand level increments to individual diameter increments, with null increments for trees below a threshold circumference value and increments increasing linearly with tree circumference above this threshold (Appendix S3). The parameters of this empirical function vary dynamically according to the dendrometric characteristics of the pixel, such as density and dominant height (height of the tallest trees). Other dendrometric variables at tree level and pixel level are then derived from diameter growth. The growth model was calibrated for trees above a certain age, namely, recruitment age (25 years for Atlas cedar). After annual tree growth, self‐thinning occurs if the number of trees exceeds the maximum number of trees in the pixel, i.e., its carrying capacity, which depends on the root mean square of circumferences. Self‐thinning is a selective mortality process where the smallest trees are progressively eliminated one by one until reaching a relative density index value below the maximal density, following a relation calibrated for Atlas cedar (Courbet, 2002).

Adding phenotypic variation of a growth‐related trait in the deterministic growth model has an impact on predicted tree growth that varies dynamically during stand development, depending on competition in a complex way (detailed in Appendix S4). As a general trend, the phenotypic variation in vigor does not change the predicted mean annual growth and mean diameter until selective self‐thinning mortality occurs, but it increases these predicted means when self‐thinning occurs. This consequence of self‐thinning relates to the fact that phenotypic variation in vigor globally increases the predicted variance of annual growth and diameter, thus increasing the predicted mean of trees that survive to selective self‐thinning and increasing the selection differential. Competition without mortality inflates the impact of phenotypic variation in vigor on predicted variances of annual growth and diameter, but selective mortality reduces this impact.

#### 2.1.4 Regeneration model

Trees are considered fertile if they are older than the recruitment age and have a diameter above a threshold diameter value (15 cm for Atlas cedar). The annual production of cones by a female tree is determined as an ordinal variable related to tree diameter and crown height with inter‐annual (for each tree) and inter‐individual (among equal‐size trees) stochasticity; this relationship and the mean number of viable seeds per cone were calibrated on Atlas cedar (Appendix S3). Individual male fertility is a relative index based on the square of tree diameter as a proxy of pollen production.

For regeneration, single seeds and pollen grains are not simulated per se, but parents are assigned to each establishing seedling as a function of their fecundity and dispersal processes. By default, the number of seedlings established in a pixel is arbitrarily scaled to the predicted seed rain received in 1 m^2^ at the center of the pixel from all mother trees of the stand, which ends in the range of seedling density values observed in Atlas cedar regeneration plots (this can be changed by the user, see further discussion in Appendix S2B).

The composition of the seedling cohort in each pixel is determined by the contribution of all mother‐trees, given their female fecundity and their distance to the center of the pixel following a 2D‐exponential fat‐tailed and long‐distance dispersal kernel calibrated for Atlas cedar seeds with mean dispersal distance 240 m (E. Klein (personal communication, 2019)). Once a seedling is established and its mother‐tree assigned, its father‐tree is determined among the fertile trees with a probability that may represent different types of mating systems, with or without selfing allowed. Here, the probability of each fertile tree to be the father of a seedling depends on its male fertility only. The genotype of each seedling is then derived from the genotypes of its parents, accounting for stochastic gamete sorting and recombination. Since this is a finite‐loci model to simulate short‐term evolution, i.e., only a few generations of trees, mutations are neglected.

Each new seedling is initialized with a diameter drawn from the expected distribution of diameter at recruitment age given tree density, and it does not grow until reaching this age. Since the growth model is calibrated for even‐aged cohorts, adult trees that may eventually remain in the pixel are removed when recruitment starts to let the recruited seedlings grow. Self‐thinning operates to regulate the density of recruited seedlings, and the age of all accumulated seedlings over the regeneration period is set to the recruitment age. Note that the initial diameter of a new seedling is drawn at random, but its genotypic value for vigor is derived from the parents. Thus, the genotype only starts to influence tree diameter after the first step of growth. In other words, the inter‐individual variation resulting from pre‐recruitment processes is considered to be neutral with regard to the quantitative traits in the model and genetic variation only affects post‐recruitment processes.

#### 2.1.5 Random mortality through disturbance

In Luberon2, stochastic disturbances are simulated through their quantitative impacts on the demographic processes of growth, survival and/or reproduction. The disturbance regime of impacts is characterized by the distribution of impact intensities, their frequency of occurrence and, eventually, spatial patterns of impacts. The regime of disturbance impacts can be calibrated on empirical surveys. Here, we considered stochastic disturbances killing trees randomly, regardless of their individual characteristics. Each year, a potential mortality rate is drawn for the whole stand, which is then locally reduced in pixels where trees are fewer and/or smaller according to the sum of individual crown height. This type of density‐dependent stress level can be observed with abiotic stresses as well as biotic interactions. Additionally, the model allows further adjustment of the probability of death at the individual tree level based on genetic sensitivity to this disturbance, an option that is not used in the following simulations.

Here, we considered three disturbance regimes of random mortality. We simulated a reference scenario with no disturbance at all. A medium disturbance regime was obtained by drawing the annual potential mortality rate from a unimodal Weibull distribution resulting in an average annual mortality rate of around 2%, which corresponds to observed rates in other tree species (Petit‐Cailleux et al., 2021). A severe disturbance regime was obtained by drawing the annual potential mortality rate from a bimodal Weibull distribution of frequent low‐mortality events and rare high‐mortality events (low‐mortality events were five times more frequent than high‐mortality events), reaching an average annual mortality rate around 12%.

#### 2.1.6 Silvicultural interventions and scenarios

Luberon2 implements various types of silvicultural interventions (called “harvest” when all the trees are removed and “thinning” when only some trees are removed). Thinning criteria can be defined at the stand level based on target tree density or target basal area with thinning from below (i.e. removing smaller trees), or thinning from above (removing bigger trees), or at random thinning. Alternatively, criteria can be defined at the individual tree level, by age, size, spatial criteria or any combination thereof. In this study, all interventions were random and thus selectively neutral.

The simulations were initialized with a 25 year old Atlas cedar forest, consisting of one 4.41 ha environmentally homogeneous stand with a density of 2800 trees/ha, and a site index of 20 m at 50 years. We applied seven silvicultural scenarios during four successive cycles repeating the same sequences of interventions with non‐overlapping generation turnover (Appendix S1). The silvicultural scenarios differed in terms of cycle length (60 or 100 years), time and intensity of thinning interventions (Table 1). For all scenarios, each management cycle (including the fourth) ended with 3 years of regeneration triggered by an intervention called the “seeding cut”, leaving 110 seed‐trees/ha. At the end of these 3 years, the final harvest removed the seed trees to leave only new seedlings. The number of created seedlings was determined by the regeneration model, but we reduced this number at the age of recruitment to reset the initial value of 2800 trees/ha (using random thinning) to avoid any effect of demographic stochasticity at the regeneration step blurring the impact of silviculture interventions. In two unthinned scenarios (named *U‐long* and *U‐short* for long or short cycles, respectively), we only applied the seeding cut and the final harvest to allow regeneration, control the cycle length and align the number of seed‐trees contributing to the regeneration with the other scenarios. As baseline scenarios for long and short cycle management (*B‐long* and *B‐short*), we followed silviculture guidelines for Atlas cedar in France (Ladier et al., 2012; and CNPF, 2020, respectively). To disentangle the effects of intensity and frequency of thinning, we also derived three exploratory scenarios from the *B‐long* scenario: low intensity (*E‐low*), delayed (*E‐delayed*) and relaxed (*E‐relaxed*). For *E‐low*, we performed all thinning with higher target density values to maintain a higher stand density than in the reference. For *E‐delayed*, we skipped the first three thinning and kept the remaining thinning with the same target density as in the reference. For *E‐relaxed*, we skipped the second to fourth thinning (Table 1).

**TABLE 1:** Sequences of thinning in the different silvicultural scenarios: stand age at intervention and, in brackets, target tree density after the intervention.

Scenario	Thinning 1	Thinning 2	Thinning 3	Thinning 4	Thinning 5	Thinning 6	Seeding cut	Final harvest
U‐long: Unthinned, long cycle	–	–	–	–	–	–	100 (110)	103 (0)
U‐short: Unthinned, short cycle	–	–	–	–	–	–	60 (110)	63 (0)
B‐long: Baseline thinning, long cycle	25 (1100)	40 (600)	50 (430)	60 (320)	70 (245)	80 (200)	100 (110)	103 (0)
B‐short: Baseline thinning, short cycle	25 (1100)	30 (600)	40 (200)	–	–	–	60 (110)	63 (0)
E‐low: Exploratory low intensity thinning, long cycle	–	40 (2400)	50 (1720)	60 (1280)	70 (980)	80 (800)	100 (110)	103 (0)
E‐delayed: Exploratory delayed thinning, long cycle	–	–	–	60 (320)	70 (245)	80 (200)	100 (110)	103 (0)
E‐relaxed: Exploratory relaxed thinning, long cycle	25 (1100)	–	–	–	70 (245)	80 (200)	100 (110)	103 (0)

*Note*: Each cycle ends with a seeding cut harmonizing the density of seed trees over all scenarios, and a final harvest of seed trees after 3 years of reproduction. In the four cases where thinning 1 is omitted, seedling density is nevertheless reduced at age 25 to the same value as initialization, i.e., 2800 trees/ha, for harmonization. All interventions are made randomly with no tree choice.

#### 2.1.7 Genetic model

Luberon2 uses a finite‐loci quantitative genetic model to represent within‐stand phenotypic variation, using the Genetics Library of Capsis (Seynave & Pichot, 2004). Nuclear diploid individual genotypes are defined along a genetic map, which contains multi‐allelic neutral loci (not used here) and diallelic QTL controlling vigor.

Each diallelic QTL is characterized by an absolute allelic effect, with a positive value for the allele increasing the value of the trait, and the same but negative value for the alternative counterpart decreasing the value of the trait. We assume no dominance between alternative alleles, so that heterozygotes have null genotypic values. For each variable trait, the model assumes additive inheritance: the genotypic value of an individual is the sum of the effects of its alleles over the QTL controlling the trait. At each simulation step (year), the individual phenotypic values are computed as the sum of the genotypic value with an individual environmental term made of two components: a fixed component and an “inter‐step” component that randomly varies annually. The genetic mean and variance of the population are computed each year from the individual genotypic values.

Here, we used a heuristic algorithm to produce the initial genotypic data with the following sets of target genetic parameters (more information on this algorithm in the Luberon2 user manual). Individual vigor, i.e., the individual deviation to the baseline prediction of individual diameter increment as previously defined, was the only variable trait. The response to selection depends on the initial genetic variance and, for a given initial variance, changes in genetic mean and variance depend on the genetic architecture of the trait, defined by the number of QTL, the distribution of allelic effects, and the allele frequencies. To test the sensitivity of the model to assumptions made on the genetic architecture of the variable trait (the genetic architecture is generally unknown), we fixed the initial additive variance of vigor and considered two contrasted genetic architectures, either 10 or 50 QTL, which are expected to result in slightly different micro‐evolutionary outcomes (Cubry et al., 2022). We considered two cases of initial population: a reference with no phenotypic variation in vigor, i.e., no QTL effects and no environmental variance, and a panmictic population with genetic and environmental variation. In the second case, we fixed the additive genetic variance at VA = 0.0042 with a null “inter‐step” component of the environmental variance, which corresponds to the average within‐population variance estimated in 22 populations of five tree species (Fririon et al., 2023), and narrow sense heritability at *h* ^2^ = 0.3, which is usually found for growth‐related traits (Alberto et al., 2013; Cornelius, 1994). It is worth noting that, even in the case without phenotypic variation in vigor, background inter‐individual variation of tree size arises from the growth process, depending on the initial tree sizes and local growth and competition processes.

The algorithm proceeds through multiple steps to reach the target genetic variance in the initial population. First, the contributions of each QTL to the target additive variance are drawn in a gamma distribution, which results in few QTL of large contribution. Second, for each QTL, allelic frequencies are drawn in a gamma distribution. Third, the absolute allelic effects of each QTL are deduced from the contributions to the total variance and the allelic frequencies. Fourth, individual genotypes (combinations of alleles) are drawn from independent binomial distributions parameterized with the allelic frequencies. The fit of the resulting genetic mean and variance to their target value is evaluated and the successive steps are repeated until a proper fit between the realized and targeted values is reached. Because of the stochasticity involved at each of these steps, running the algorithm several times with the same target values results in different sets of allelic effects, allelic frequencies and genotype frequencies. We assume that the main driver of selection is the target variance rather than its underlying genetic setup. In the following simulations, we assessed the sensitivity of the model to this genetic initialization stochasticity by running five optimizations for each set of target genetic parameters for the scenario with non‐null genetic variance of vigor.

Finally, we used 11 initial genetic backgrounds: one reference case without phenotypic variation and 10 cases with the same additive variance, either with 10 or 50 QTL, five different genetic setups of allelic effects and frequencies in each case, with linkage equilibrium at the start.

#### 2.1.8 How selection proceeds in the demo‐genetic model

Selection proceeds through differential survival (viability selection) and contribution to regeneration (male and female fecundity selection). Natural mortality by competition, i.e., self‐thinning, is selective with regard to tree size. In this context, tree size is a key fitness component because it is the direct target of viability and fecundity selective processes. Therefore, indirect selection on vigor dynamically emerges from the model because vigor is a variable trait contributing to tree size through the growth model. The intensity of indirect selection on vigor depends on its correlation with tree size, which dynamically varies with age, competition and disturbances. More generally, in DG–ABMs, selection intensity is not a fixed parameter, it varies with population dynamics, disturbance regime and management interventions.

By contrast, in the following simulations, mortality by disturbance and by silviculture were not selective because there was no variation in sensitivity to disturbance and thinning were random. Here, the only consequence of random disturbance and random thinning was to reduce the population density, hence reducing the intensity of indirect natural selection on vigor by competition.

### 2.2 Simulation plan and analysis of simulation outputs

The full‐factorial design combining 11 genetic backgrounds with seven silvicultural scenarios and three disturbance regimes resulted in 231 scenarios that were replicated 10 times to account for stochasticity in the regeneration compartment of the model, for a total of 2310 simulation runs.

For each simulation run, i.e., four successive cycles of the same silvicultural regime, the dendrometric and genetic variables were stored annually (Table 2). Annual changes within a cycle informed on the mechanisms that progressively generate the selection differential between the subset of reproductive trees and the original population, while inter‐generational changes also accounted for the effect of mating success and heredity processes. We computed the average evolutionary rate per generation for vigor over the whole period of time, *H*_0_, defined by Gingerich (1993, 2009) as:

$$H_{0}=\left({\text{zdiff}}_{i}/\sqrt{\text{zvar}.w_{i}}\right)/n.\mathrm{gen}$$

where zdiff_*i*_ is the observed change in the mean value of the trait in a temporal interval *i*, zvar.*w*_i_ is the pooled within‐population phenotypic variance of the trait sampled at the beginning and at the end of the temporal interval *i*, and *n*.gen is the number of generations. The temporal interval that we used is defined by the beginning of the first silvicultural cycle and the regeneration produced after the fourth, corresponding to four complete generations.

**TABLE 2:** Description of the Luberon2 output variables analyzed in this study.

Output variable	Code	Unit	Description
Number of trees per hectare	*Nha*	–	The number of alive individual trees per hectare
Population genetic mean of vigor	*μG.Vig*	cm.year^−1^	The population genetic mean of vigor
Additive genetic variance of vigor	*VA(Vig)*	cm^2^.year^−2^	The variance of individual vigor genetic values within the population
Heterozygosity	*He*	–	Nei's expected heterozygosity
Total timber volume produced per hectare	*VhaProd*	m^3^.ha^−1^	The cumulative volume of timber produced per hectare over each cycle, including the volume of dead trees
Quadratic mean diameter	*QMD*	cm	The diameter of the tree with an average square diameter
Self‐thinning‐induced deaths	*DeadS‐T*	–	Number of deaths by self‐thinning per hectare over each cycle
Disturbance‐induced deaths	*DeadD*	–	Number of deaths by disturbance per hectare over each cycle

Here, there is typical eco‐evolutionary feedback: selective competition on tree size generates an indirect selection on vigor, and the genetic gain on vigor may increase tree size and thus increase selective competition intensity. We studied these feedback effects in a scenario with no silviculture. We first analyzed the effects of genetic evolution on forest productivity by computing the total volume of timber produced per hectare through the years (*VhaProd*), which includes the volume of dead trees. We then followed the evolution of the quadratic mean diameter (*QMD*) as a summary variable of the distribution of tree size (Curtis & Marshall, 2000), which only considers the surviving trees, and therefore, accounts for the progressive establishment of the selection differential.

## 3 RESULTS

### 3.1 Natural selection on vigor through differential mortality and reproduction in the unthinned scenario without disturbance

**FIGURE 2:**
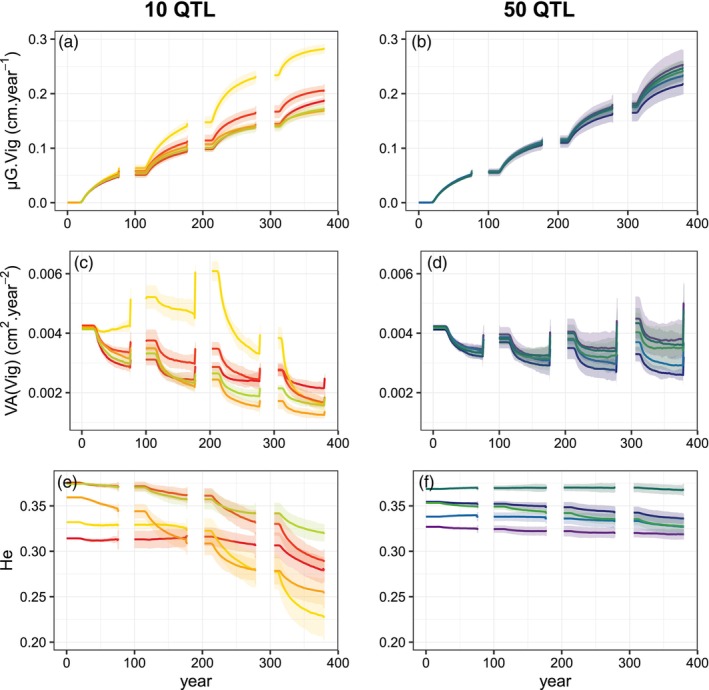
Dynamics of population genetic parameters in the unthinned long cycle scenario (U‐long) without disturbance for 10 QTL and 50 QTL (in columns): population genetic mean, *μG.Vig* (a, b); genetic variance, *VA(Vig)* (c, d) and QTL gene diversity, *He* (e, f). The different colors indicate five genetic setups, i.e., combinations of allelic effects and allelic frequencies representing the same target additive genetic variance, for each number of QTL. The shaded areas represent the 95% intervals over 10 replicates for each genetic setup. Trees are 25 years old when simulations start at Year 0. The pre‐recruitment period is deliberately not represented because the phenotype is not considered in the model during this phase.

**FIGURE 3:**
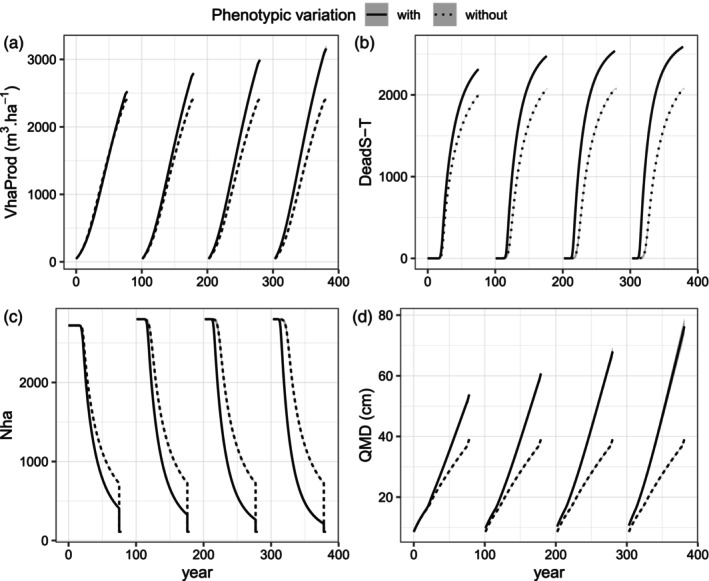
Dynamics of forest productivity, demography and tree size in the unthinned long cycle scenario (U‐long) without disturbance, with or without phenotypic variation (line types): (a) total timber volume produced per hectare (VhaProd); (b) number of deaths by self‐thinning per hectare, *DeadS‐T*; (c) number of trees per hectare, *Nha*; (d) quadratic mean diameter, QMD. This figure illustrates one genetic setup with 50 QTL; see Appendix S5 Figure S3 for the other genetic setups. The 95% intervals over 10 replicates, represented by shaded areas, are imperceptible. Trees are 25 year old when simulations start at Year 0. The pre‐recruitment period is deliberately not represented because the phenotype is not considered in the model during this phase.

There were enough reproducing trees to prevent genetic erosion by genetic drift on the short‐temporal scale of these simulations (data not shown). We first characterized the process of natural density‐dependent soft selection on vigor that emerged from the model in the unthinned long cycle scenario (*U‐long*) without disturbance. Over four consecutive cycles, the genetic mean of vigor continuously increased, both within‐ and across generations (Figure 2a,b). The evolution of genetic variance resulted from the combined effect of changes in QTL allele frequencies and selection‐induced linkage disequilibrium generating negative covariance among QTL (data not shown), the so‐called Bulmer effect (Bulmer, 1971), with alternance of reduction (within‐generation) and restoration (at reproduction) phases (Figure 2c–f). Within each generation, once self‐thinning started, viability selection proceeded continuously with annual changes of the genetic mean and variance of vigor, with a progressive decrease in the rate of change of the mean. This progressive decrease of evolutionary changes resulted from the combined effects of three mechanisms: (i) the reduction of standing variance available for selection, which progressively reduced the response to selection; (ii) the decrease in mortality rate by self‐thinning while population density decreased (Figure 3a,b), which progressively reduced selection intensity and (iii) the progressive increase in the correlation between vigor and tree size with age (data not shown), which increased the strength of indirect selection on vigor. At each regeneration step, sexual reproduction restored the genetic variance by reducing linkage disequilibrium among QTL and generated a high density of young trees, thus restoring the dynamics of selective competition. In these simulations, fecundity selection only occurred during the 3 years of reproduction at the end of each cycle.

With 10 QTL, the average increase of the genetic mean was lower than with 50 QTL, and the heterogeneity among genetic setups was higher (Figure 2). Furthermore, the reduction of the genetic variance and QTL gene diversity (*H*_*e*_) was systematically more pronounced with 10 QTL than with 50 QTL. The variation among 10 replicates with the same initial genetic setup, resulting from stochasticity in the reproduction process, was negligible except for the predicted changes in genetic variance with 50 QTL, and, in this case, it remained much less than the variation due to assumptions on genetic architecture (Figure 2).

In the next sections, we focus on one setup of the 50 QTL case; the results obtained with all genetic backgrounds were consistent with this particular case and are provided in Appendix S5.

### 3.2 Feedback effect of genetic evolution on population productivity and tree size in the unthinned scenario without disturbance

We explored the feedback effect of genetic evolution across generations on stand productivity and tree size in the unthinned long cycle scenario (*U‐long*) without disturbance by comparing simulations with vs without phenotypic variation in vigor. Stand productivity was higher with phenotypic variation than without. At the end of the first generation, total timber volume produced was 5% higher with phenotypic variation than without, due to the progressive elimination of the least vigorous individuals (Figure 3a). Then, genetic gain in vigor increased the whole‐cycle timber volume produced by 8% per generation on average. Genetic evolution in vigor increased stand productivity by two mechanisms. First, higher mean vigor increased annual productivity before self‐thinning regulation, as evidenced by a higher level of self‐thinning regulation with genetic evolution than without (Figure 3b). Second, higher mean vigor enabled the population to reach its maximum productivity faster, as evidenced by earlier self‐thinning in the advanced generations with genetic evolution than without (Figure 3b,c).

The effect of genetic evolution on the size of surviving trees was higher than on whole stand productivity, and the difference in tree size at the end of each cycle between scenarios with or without evolution illustrates the selection differential (Figure 3d). At the end of the first generation, quadratic mean tree diameter was 38% higher with phenotypic variation than without. The presence of phenotypic variation in vigor progressively increased variance in tree diameter within each silvicultural cycle, and the selective elimination of less vigorous individuals before reproduction led to a progressive increase in tree size across generations, i.e., a genetic improvement process, such that the QMD increased by 12% per generation on average (Figure 3d). As an eco‐evolutionary feedback loop, the genetic evolution of tree vigor progressively led to more early and more intense selective competition processes across generations (Figure 3b,c), which reinforced selection for vigor. In other words, the selection differential increased with the mean vigor of the population.

### 3.3 Impacts of thinning scenarios and disturbance regimes on natural selection and evolutionary rates

**FIGURE 4:**
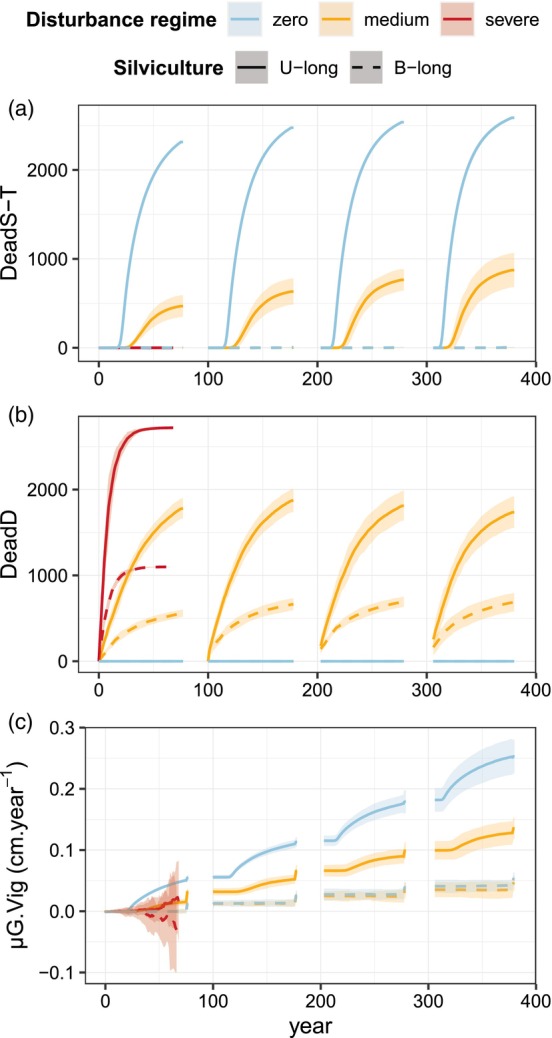
Dynamics of mortality and genetic changes in the long cycle scenarios with phenotypic variation, unthinned (*U‐long*) or with baseline thinning (*B‐long*) and three disturbance regimes (in colors): (a) deaths by self‐thinning, *DeadS‐T*; (b) deaths by disturbance, *DeadD*; (c) population genetic mean of vigor, *μG.Vig*. This figure illustrates one genetic setup with 50 QTL; see Appendix S5 Figure S4 for the other genetic setups. The shaded areas represent the 95% intervals over 10 replicates for each scenario. With the severe disturbance regime, the population collapsed during the first cycle. Trees are 25 year old when simulations start at Year 0. The pre‐recruitment period is deliberately not represented because the phenotype is not considered in the model during this phase.

We compared simulations implementing different silvicultural scenarios (Table 1) and disturbance regimes (null, medium, high) to investigate their joint impacts on the natural selection process and overall evolutionary rates. In all cases, we included phenotypic variation in vigor. In the unthinned long cycle scenario (*U‐long*) without disturbance, selective self‐thinning was the only mechanism of mortality (Figure 4a,b), and viability selection by competition was the main driver of genetic evolution in vigor, with the negligible additional effect of fecundity selection (Figure 4c). In the baseline long cycle scenario with thinning (*B‐long*), the non‐selective decrease of tree density reduced the rate of mortality by self‐thinning (Figure 4a), thus reducing the intensity of viability selection to a negligible level. The only mechanism of selection was fecundity selection causing qualitative leaps in mean vigor at the time of reproduction (Figure 4c). Similarly, disturbance acted as another form of non‐selective mortality reducing population density and competition intensity (Figure 4b). With the medium disturbance regime, self‐thinning persisted in the unthinned long cycle scenario but was reduced by 73% on average and, consequently, the selection differential on vigor, i.e., the difference between the genetic mean of seed trees and that of the initial population at the beginning of the cycle, was reduced by 63% on average. In the baseline long cycle scenario with thinning with a medium disturbance regime, viability selection by self‐thinning did not occur anymore (Figure 4). The severe disturbance regime caused a population collapse at the end of the first generation.

**FIGURE 5:**
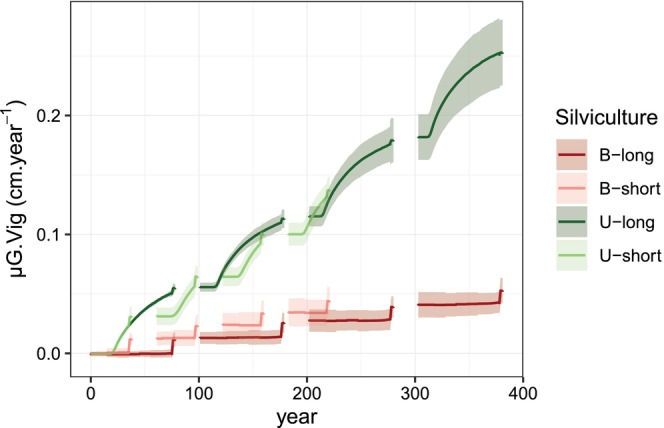
Dynamics of the population genetic mean of vigor (*μG.Vig*) in four scenarios, long or short cycle unthinned (*U‐long*, *U‐short*, respectively, in colors) or with baseline thinning (*B‐long*, *B‐short*, respectively). This figure illustrates one genetic setup with 50 QTL; see Appendix S5 Figure S5 for the other genetic setups. The shaded areas represent the 95% intervals over 10 replicates for each scenario. Trees are 25 years old when simulations start at Year 0. The pre‐recruitment period is deliberately not represented because the phenotype is not considered in the model during this phase.

Comparing unthinned scenarios with long or short cycles (*U‐long* and *U‐short*, respectively), viability selection by competition was the main evolutionary force increasing population genetic mean in both cases. The short cycle induced shorter exposure to selective competition, concentrated in early stages when competition was maximum, but more opportunities for fecundity selection, finally resulting in a slightly lower genetic mean at most dates (Figure 5). By contrast, in the baseline scenarios with thinning (*B‐long* and *B‐short*), random thinning impeded viability selection by competition, and fecundity selection remained the only driver of changes in population genetic mean (Figure 5). In this case, the short‐cycle scenario having more frequent reproductive events than long‐cycle scenarios also reached a higher genetic mean at a given date (Figure 5), despite a lower evolutionary rate per generation (see below). Thus, the impact of the length of silvicultural cycles depended on the importance of viability selection by competition, i.e., on the relative roles of selective self‐thinning or random thinning by management.

**FIGURE 6:**
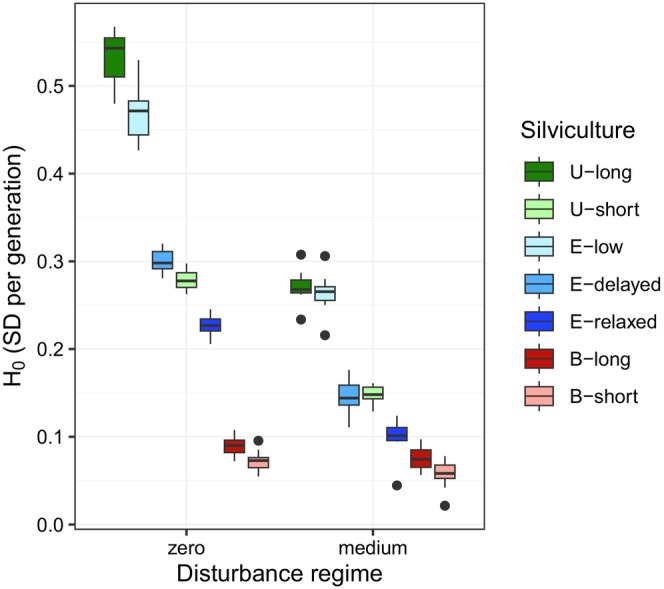
Average evolutionary rate (*H*_0_) in vigor after four generations for the different silvicultural scenarios (in colors) and disturbance regimes (x‐axis, the severe disturbance regime is not represented because it led to population collapse). The boxplots illustrate the distribution of values over 10 replicates in each case. This figure illustrates one genetic setup with 50 QTL; see Appendix S5 Figure S6 for the other genetic setups. Silvicultural scenarios: unthinned long cycle (U‐long); unthinned short cycle (U‐short); baseline thinning long cycle (B‐long); baseline thinning short cycle (B‐short); exploratory low‐intensity thinning long cycle (E‐low); exploratory delayed thinning long cycle (E‐delayed); exploratory relaxed thinning long cycle (E‐relaxed).

More intensive random thinning (from unthinned to exploratory and baseline thinning scenarios, from long‐to‐short cycles) or more intensive disturbance regimes were associated with higher reduction in selective competition intensity and, consequently, with lower evolutionary rates (Figure 6). The unthinned long‐cycle scenarios (*U‐long*) resulted in the highest evolutionary rates of vigor for each disturbance regime, ranging from 0.35 to 0.62 over all simulation runs when there was no disturbance, depending on the number of QTL and the genetic setup (Appendix S5). In comparison, the exploratory low‐intensity thinning scenarios (*E‐low*) reduced the evolutionary rate by 9% on average over the disturbance regimes, and other scenarios resulted in higher reduction, in the following order: exploratory delayed thinning scenarios (*E‐delayed*, by 45%), unthinned short cycle scenarios (*U‐short*, by 47%), exploratory relaxed thinning scenarios (*E‐relaxed*, by 59%), baseline long cycle scenarios (*B‐long*, by 80%) and finally baseline short cycle scenarios (*B‐short*, by 84%). On average over all the silvicultural regimes, the medium disturbance regime reduced the evolutionary rates by 46%. The severe disturbance regime caused a population collapse that did not allow any genetic evolution.

## 4 DISCUSSION

This study highlights the power of demo‐genetic models to investigate the interactions between evolutionary and demographic processes: how demographic processes including management drive predicted evolutionary rates and, vice‐versa, how evolutionary processes drive predicted population growth and dynamics. Here, we implemented this approach by integrating genetic variation of vigor in an individual‐based forest growth model, wherein a trait directly linked to the demographic processes also drives individual fitness components (i.e., growth, survival and fecundity). This represents a situation of particularly strong interaction between evolutionary and demographic processes, and we expect less interaction when the variable traits are less tightly connected to demographic processes.

### 4.1 Predicted evolutionary rates and sensitivity to genetic assumptions

Considering a plausible level of initial additive variance and various scenarios of silviculture and disturbance regimes, the predicted evolutionary rates of vigor over four generations (from 0 to 0.62 over all simulations) were in the range of published empirical estimates for wild plant and animal populations: from 0 to 0.65 (Bone & Farres, 2001) and 0.05 to 0.44 (Bonnet et al., 2022; Gingerich, 2009), respectively. Therefore, we consider that Luberon2 can reasonably be used to characterize and quantify the eco‐evolutionary feedbacks that emerge from demo‐genetic coupling in the time frame of a few generations, as we did in this study. However, elaborating longer‐term predictions would require accounting for the consequences of interactions between evolutionary and demographic processes that may progressively deviate tree growth predictions from realistic values after several generations of selection (e.g., by adding constraints on the long‐term response to selection in the model).

The evolutionary rate does not only depend on the standing variation and heritability but also the genetic architecture of the trait. Here, we investigated the sensitivity of model predictions to the number of QTL and to the distribution of allelic effects among QTL. The evolutionary rate was higher, and the reduction of additive variance and QTL diversity was lower, with 50 QTL than with 10 QTL. This difference may be attributable to the higher contribution of covariance between QTL to the selection response with 50 QTL than with 10 QTL (Cubry et al., 2022; Le Corre & Kremer, 2012). The evolutionary predictions of the model were much less sensitive to the distribution of allelic effects and allelic frequencies with 50 QTL than with 10 QTL. Furthermore, our results suggest that the comparisons among silviculture and disturbance scenarios (discussed below) are qualitatively robust to the assumptions about the genetic architecture of the trait of interest. In any case, since the genetic architecture of traits is rarely known in detail, simulation studies based on DG–ABM may account for the sensitivity of the model to genetic architecture assumptions by including several genetic makeup scenarios in the simulation plan.

### 4.2 Predicted tree growth improvement by density‐dependent soft selection

In our simulations, natural density‐dependent soft selection on vigor increased QMD by 12% per generation over four generations, which is comparable to the estimated genetic gain for growth observed in classical breeding programs (e.g., 10%–25% according to Jansson et al., 2017).

Selection is a dynamic process, and its intensity varies with stand development. In a baseline scenario without thinning and without disturbance, several mechanisms occur concurrently within each cycle: (i) the mortality rate by self‐thinning progressively decreases while population density decreases, which in turn decreases the selection intensity; (ii) genetic variance in vigor progressively decreases due to selective mortality, which decreases the response to selection; (iii) the correlation between vigor and tree diameter, a major component of fitness, progressively increases with age, which increases the selection gradient on vigor. In our simulations, the third mechanism did not compensate for the other two, and the higher rate of change in the genetic mean and variance was observed at a very early stage, 1 or 2 years after the start of self‐thinning mortality.

The model did not account for possible selection on vigor during the juvenile phase before recruitment age because the initial diameter was randomly distributed among trees regardless of their genotypes at this age. Selection on vigor only operated once a correlation between vigor and tree diameter was established and competition‐induced selective mortality started. However, it has been shown that selection can start during this juvenile stage, in particular, due to the elimination of inbred individuals (e.g., Finkeldey & Ziehe, 2004; Skrøppa, 1994). Juvenile selection will deserve further attention in future developments of the model. However, the exclusion of inbreeding depression effects on growth in our model here at least partly compensates for neglecting juvenile selection.

Various patterns or processes not included in the model might reduce the intensity of selection on vigor compared with these simulations. For instance, environmental constraint limiting the phenotypic expression of vigor, within‐stand heterogeneity of disturbance regime, or phenotypic trade‐off between vigor and sensitivity to stress would reduce the correlation between vigor and tree size, thus reducing the intensity of indirect selection on vigor (Fririon et al., 2023). Resource allocation trade‐off between growth and reproductive functions (Wardlaw, 1990) or life history trade‐off between growth and longevity (Roskilly et al., 2019) would reduce the synergy of viability selection and fecundity selection on vigor. Temporal changes in the ranking of genetic values with age, which occurs for growth‐related traits when there is genetic variation in growth kinetics (Danjon, 1994), would also reduce the response to selection. The ultimate consequences of all these patterns and processes on the correlation between vigor and fitness, i.e., the selection gradient, are hardly predictable because the selection gradient dynamically results from complex interactions between multiple processes. Therefore, we consider that the evolutionary rates obtained in this study represent an upper bound, but still plausible, intensity of natural density‐dependent soft selection on vigor.

### 4.3 Impacts of neutral disturbance and management on natural selection

A major result of this study is that non‐selective demographic events can indirectly have major evolutionary consequences through their impact on density‐dependent soft selection processes. In particular, random thinning reduced the intensity of competition‐induced selection for vigor. But this result can be generalized: for any kind of trait under natural selection, management interventions that exclude individuals from the natural selection process tend to interfere with natural selection.

In these simulations, disturbance and thinning were neutral with regard to vigor, as mortality induced by these events was independent of vigor. By reducing competition intensity, neutral disturbance and thinning reduced the intensity of natural viability selection due to competition, without directly affecting the intensity of fecundity selection (except that relaxing viability selection slightly increased the standing variance when fecundity selection occurs). Thus, compared to the baseline unthinned scenario without disturbance, the rate of evolution was reduced by disturbance alone, depending on the disturbance regime, and reduced by management alone, depending on the silvicultural scenario. When both disturbance and management were combined, the rate at which vigor evolved was drastically reduced. There was no synergy but rather a negative interaction effect so that the impact of disturbance on the rate of evolution was reduced in the scenarios with management and vice‐versa.

In reality, the negative impact of thinning on the intensity of natural selection for vigor can be reinforced or balanced by the forester's tree choice, i.e., when non‐random thinning is applied, either “from below” (smaller trees removed preferably) or “from above” (bigger trees removed preferably), at each step. Therefore, the evolutionary impact of real forest management combines both effects: the regulation of natural evolutionary processes combined with direct or indirect anthropogenic selection, which can ultimately result in higher rates of evolution in human‐impacted contexts than in wild populations as observed in animal populations (Hendry et al., 2008). Various types of tree choice can easily be simulated with Luberon2, but addressing this management‐induced selection requires an appropriate design to cover the broad range of possible silvicultural management strategies. Similarly, the negative impact of random disturbance may be reinforced or balanced in the case of size‐dependent disturbance.

Our results show that shortening the silvicultural cycle has multiple impacts on natural selection. First, it increases the frequency of fecundity selection opportunities, with evolutionary consequences that depend on the changes in correlation between vigor and fecundity throughout the silvicultural cycle. Second, it shortens the exposure period to competition, with impacts on viability selection that depend on the competition intensity over the remaining exposure period, but it concentrates competition on the early stage when competition is at maximum. In our simulations, shortening the silvicultural cycle globally reduced the period of time during which viability selection occurred and the associated genetic gain because self‐thinning started late (at age 43) and because vigor had no effect on competing ability before the recruitment age of 25. However, in reality, the evolutionary consequences of shortening the silvicultural cycle would be very sensitive to the intragenerational demographic dynamics, which are complex and multifactorial, depending on initial tree density, local site fertility, population vigor and thinning and disturbance regimes.

### 4.4 Monitoring genetic changes within and across generations

This study highlights the need to compare genetic mean and variances between generations at similar life stages, as the annual fluctuations in genetic mean and variances within each generation may not faithfully represent the effective evolutionary signal passed from one generation to the next. For the genetic mean of vigor, viability and fecundity selection mechanisms operated in the same direction, with a proportionally higher impact of the former in our stand density conditions, resulting in a positive correlation between within‐generation changes and inter‐generational evolution. The situation was radically different for genetic variance. The genetic variance was restored at each reproduction event after regular reduction due to linkage disequilibrium through the Bulmer effect during the viability selection process within the previous generation. This alternation of reduction and restoration phases resulted in weak inter‐generational evolution of variance despite intensive selection and a decrease of variance within each cycle. The restoration of variance at reproduction was more efficient with 50 QTL than with 10 QTL, which relates to a greater role of covariance among QTL with 50 than with 10 QTL as previously mentioned. The restoration of variance at each generation might be one of the reasons why standing variation is not a powerful indicator of the response to selection in wild populations (Pujol et al., 2018). For long‐lived species like trees, this also emphasizes the risk taken in synchronic empirical studies that compare different life stages at a time (e.g., adults vs. juveniles) to infer genetic evolution. Instead, indicators of evolutionary changes should be measured at equivalent stages across different generations (Hansen et al., 2012), which may be challenging for long‐lived organisms like trees.

### 4.5 Implication for forest management

In this research, each component of the demo‐genetic model had been individually calibrated on real forestry data and the simulated eco‐evolutionary feedback loops did not generate unrealistic evolutionary behavior, but the silvicultural scenarios were unrealistic because non‐selective thinning was a purely theoretical exercise to investigate the mechanisms of interaction between forest dynamics, genetic diversity and evolution, management practices and disturbances. In practice, foresters generally choose the trees they cut on diverse criteria, depending on the thinning phase within each rotation. Simulating more realistic silvicultural scenarios and assessing their genetic impacts were beyond the scope of this study. Nevertheless, a general lesson for management already taken from this study is that, in an even‐aged management system, thinning takes over the natural density‐dependent soft selection and orientates the evolution towards the forester's criteria (i.e., no selection in this work). Through the same mechanism, this study also shows that non‐selective disturbances take over density‐dependent soft selection. Shortening the rotation length has a complex effect on selection.

Extending the model to disturbance regimes with additional specific resistance traits independent from the target of density‐dependent selection would be an approach to investigate the trade‐off between resistance to disturbance and long‐term adaptation in long‐lived organisms (Schmid et al., 2022). Drawing evolutionary expectations in uneven‐aged forest systems is much more challenging because they combine specific mechanisms of asymmetrical competition, overlapping generations, and highly unbalanced male and female fecundities, which all have an effect on genetic drift and selection. Demo‐genetic models with dedicated tree growth and forest dynamics functions, e.g., forest gap models coupled with genetics, would be required for such a study. Evolutionary implications of other forestry planning practices could be assessed by extending the demo‐genetic approach to the landscape scale (Landguth et al., 2017).

## CONFLICT OF INTEREST STATEMENT

All the co‐authors disclose any potential sources of conflict of interest.

## Supporting information


Appendix S1

Appendix S2

Appendix S3

Appendix S4

Appendix S5

## Data Availability

Data and necessary elements to reproduce the research are available on the French repository for research data at https://doi.org/10.57745/DHXXBC. This archive includes all necessary elements to reproduce the research: (i) a stand‐alone version of the model; (ii) the input and output files of the simulations presented in this work; (iii) the R scripts used to analyze the simulation outputs and reproduce the figures presented in the publication.
