# Supplementary material for: A demo‐genetic model shows how silviculture reduces natural density‐dependent selection in tree populations
Source: Evol Appl. 2023 Oct 13;16(11):1830–44. doi: 10.1111/eva.13606 (PMC10681482; doi:10.1111/eva.13606)
Supplement: Supplementary file 1 — Appendix S1 [file EVA-16-1830-s001.docx]

Appendix 1 - Spatial and temporal scales in Luberon2


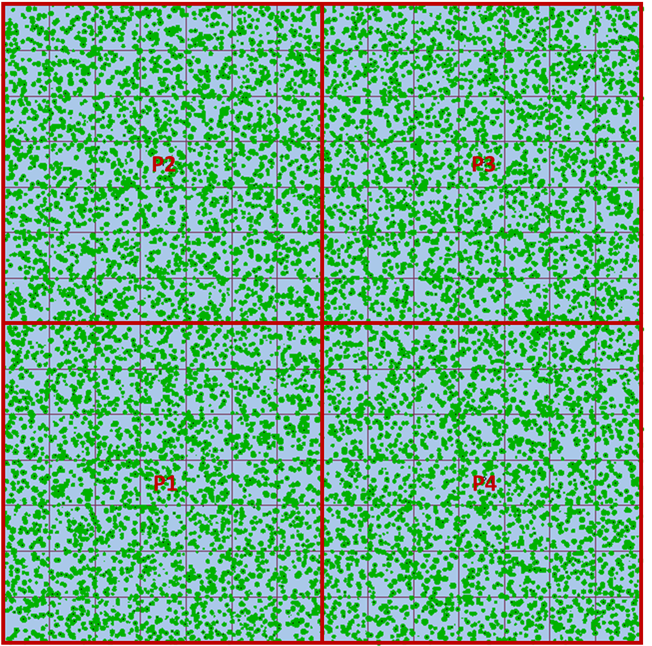


**Figure 1**: Relationship between space, environment, and demography: the simulated stand for this study consists of four parcels (P1 to P4, all with the same site index) split in regular pixels 15mx15m (background grid), starting with 12000 trees in the initial stage (green dots with a size proportional to the diameter of each tree).


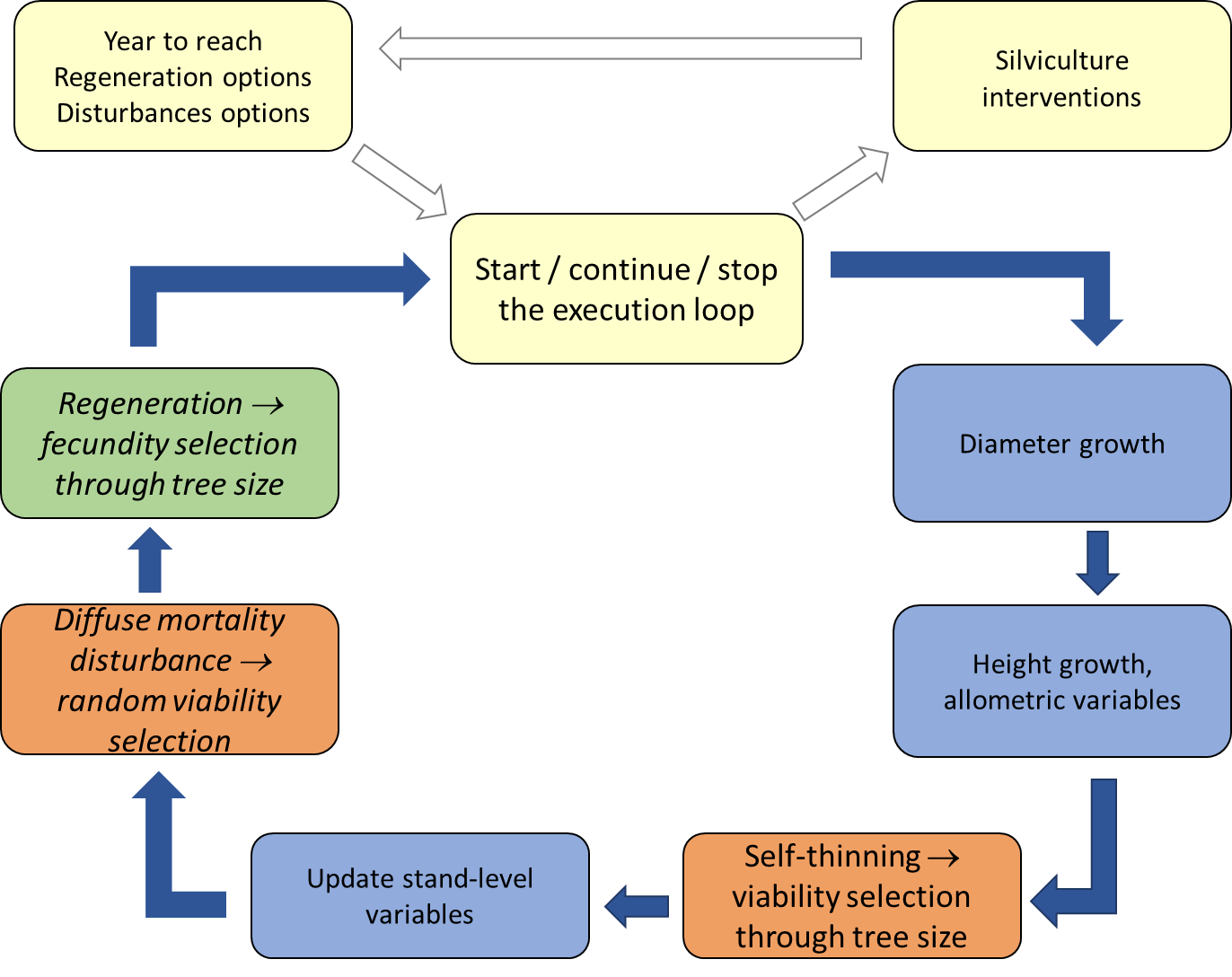


**Figure 2**: Synthetic view of the execution loop of forest dynamic processes in Luberon2. Yellow boxes represent the user’s choices, other colors are for dynamic processes (growth in blue, mortality in orange, and regeneration in green). Regeneration and disturbance (in italics) are optional depending on the user's choices.

**
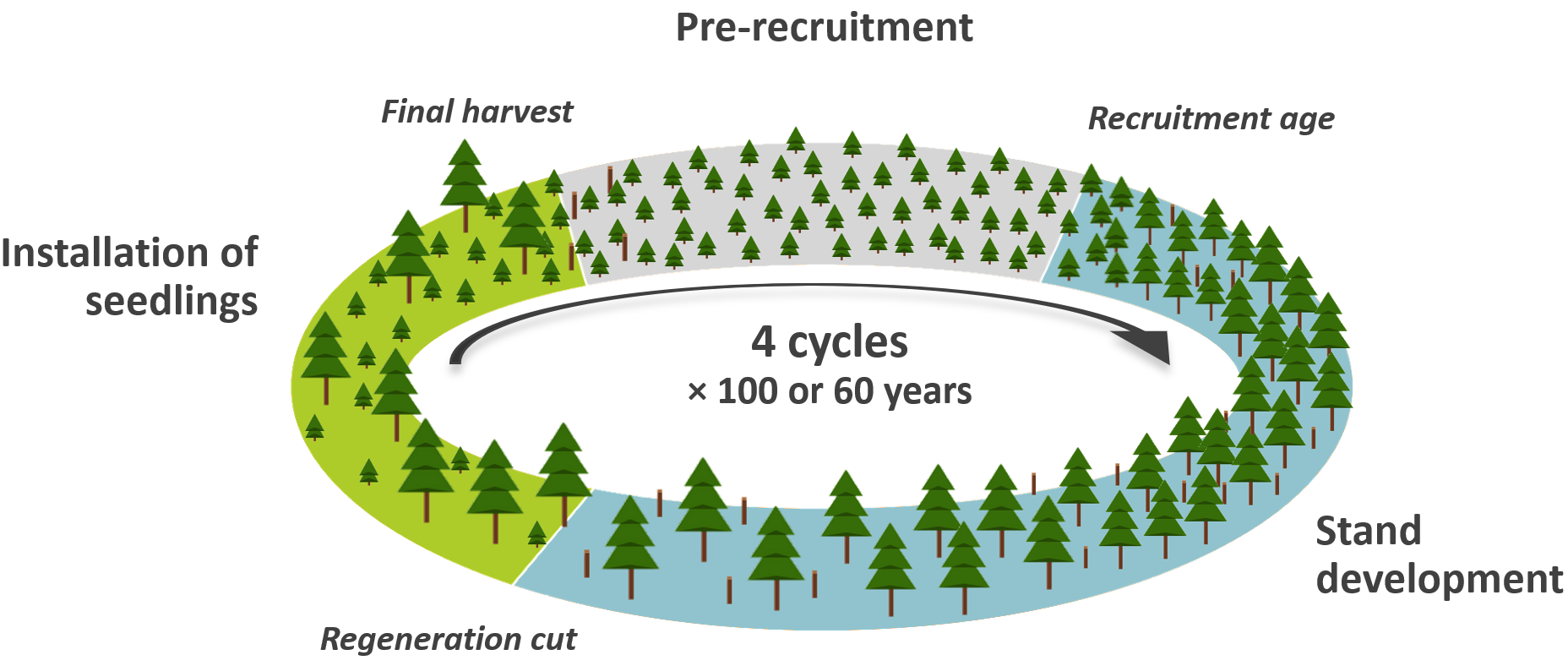
**

**Figure 3**. Chronology of the simulations. Growth and competition processes start at recruitment age (25 years for cedar). Then the diagram reads clockwise. During stand development (in blue) several thinnings are potentially carried out depending on the silvicultural scenario. When the stand is mature, at age 60 for short cycles or 100 for long cycles, a regeneration cut is carried out, and the seedlings establish over a period of three years (in green). The final harvest of the seed trees leaves only the seedlings. Before seedlings reach the recruitment age, i.e. during the pre-recruitment period (in gray), no self-thinning nor interventions take place. Each simulation run consists of four successive cycles.
