# Supplementary material for: A demo‐genetic model shows how silviculture reduces natural density‐dependent selection in tree populations
Source: Evol Appl. 2023 Oct 13;16(11):1830–44. doi: 10.1111/eva.13606 (PMC10681482; doi:10.1111/eva.13606)
Supplement: Supplementary file 2 — Appendix S2 [file EVA-16-1830-s002.docx]

Appendix 2 - Consequences of the choice of pixel size in Luberon2

# A - Consequences on stand growth and demography

## I. Global overview

Luberon2 uses a growth model calibrated for regular (even-aged) forest stands. It allows for spatially heterogeneous age structure through pixilation: pixels are regular spatial subunits that grow independently; each pixel represents an elementary even-aged growth unit. The smaller the pixels, the better the capacity to represent heterogeneous stand structure in age classes. However, decreasing the size of the growth units below the minimum size for which the growth model was originally calibrated (35mx35m for Cedrus; Courbet, 2002) induces a bias in the predictions (Sambakhe et al., 2014). In addition, as the pixel size decreases the edge effects are increased, which may create another bias in growth predictions because Luberon2 does not account for possible competition between adjacent pixels.

Therefore, we expect that pixilation may induce a bias in predicted stand-level growth variables such as basal area, diameter, or mean height, which depends on pixel size. This bias is however independent of the intensity of the interventions and disturbances, allowing for comparison of different silvicultural management and disturbance regimes.

We here quantified the sensitivity of stand-level growth and dynamics predictions due to the decrease in size of the growth units compared to the calibration. It depends on the characteristics of the inventories. The inventories are defined by the pixels composing the stand, the coordinates of the trees in the stand, the initial diameters of the trees and the initial tree density. Compared to the reference, we found that pixilation decreased the total basal area, increased mean tree size and increased stand-level mortality rate. Pixilation induced a uniformity by mortality of tree density per pixel. The uniformity of the density led to a uniformity of the growth between pixels. Pixilation therefore decreased the spatial heterogeneity of variables directly related to the number of trees, such as the total number of trees and basal area, and very slightly increased the spatial heterogeneity of mean diameters.

Considering the importance of the average deviation from the reference and the variation between replicates, we conclude that a pixel size of 15mx15m represents an acceptable compromise to allow for fine spatial age structure heterogeneity without too much bias on the predicted growth. We expect higher consequences of pixilation on stand-level growth predictions due to neglecting the competition between adjacent pixels of different ages, but these edge effects could not be quantified due to the lack of calibrated reference for such situation.

## II. Simulated scenarios

The growth model had originally been calibrated with empirical data with pixel sizes of 35mx35m minimum. We investigated the sensitivity of growth predictions for pixel sizes of 15mx15m and 10mx10m in comparison to a reference size of 60mx60m (Figure 1). We also studied the effect of pixilation on the spatial heterogeneity of growth variables.


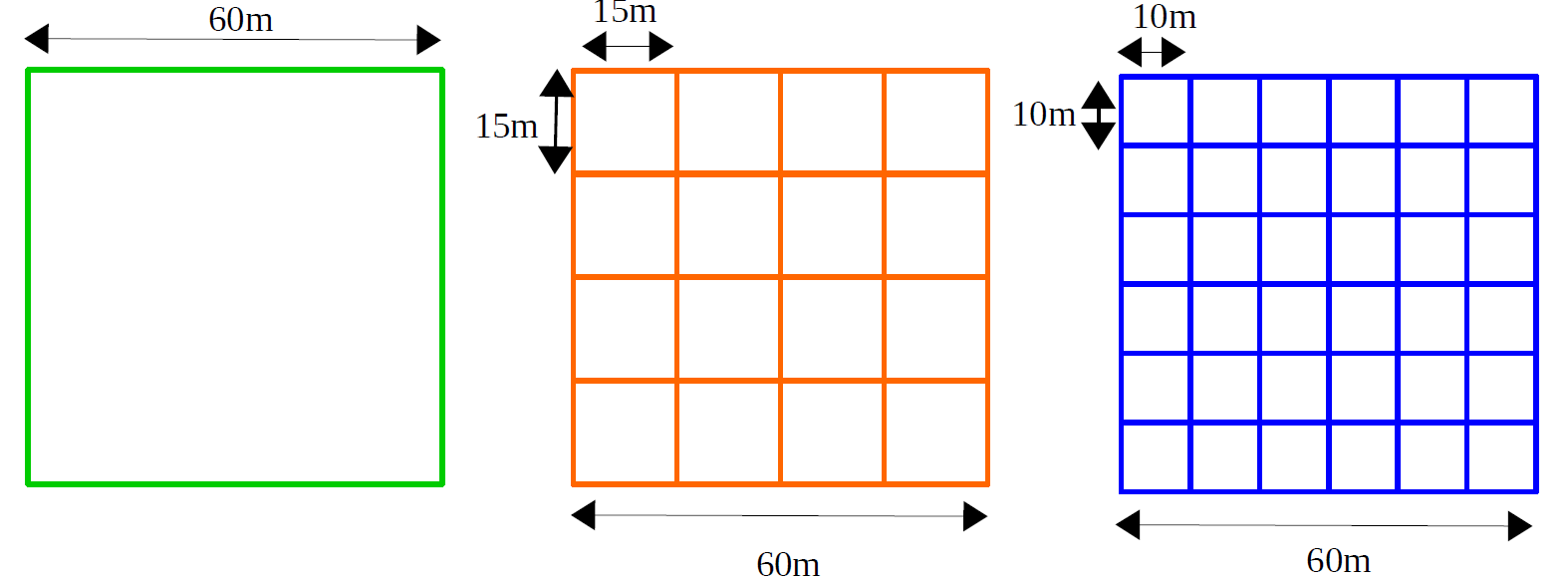


*Figure 1: Schematic of the three stand types tested: (i) a single pixel of 60mx60m, (ii) 16 pixels of 15mx15m and (iii) 36 pixels of 10mx10m*

For each pixel size, we studied the effect of three modalities: spatial tree positions, initial tree diameters, and initial tree density. Thus, we simulated nine scenarios defined in the corresponding inventory files given as input to the model:

- 60mx60m pixels – with different spatial tree positions
- 60mx60m pixels – with different initial tree diameters
- 60mx60m pixels – with different initial tree density
- 15mx15m pixels – with different spatial positions of trees
- 15mx15m pixels – with different initial tree diameters
- 15mx15m pixels – with different initial tree density
- 10mx10m pixels – with different spatial positions of trees
- 10mx10m pixels – with different initial tree diameters
- 10mx10m pixels – with different initial tree density

The density of trees, the position of trees and range of variation of initial diameters in an inventory are derived from a real empirical dataset. From the original dataset, we derived ten variations for each scenario, i.e. 90 inventories in total, as follows. For the spatial position of the trees, ten lists of x and y coordinates were randomly drawn within bounds from 1 to 59 to avoid having trees exactly on the edges. We draw ten sets of initial diameters in the same range of the original empirical dataset between 2 and 20cm. For the initial density, we used the original empirical density with 1005 trees and derived the other nine values by removing 15 trees successively. Across pixel sizes, only the number of pixels and their coordinates change, while the list of trees is the same for each condition of spatial position, initial diameter or initial density.

### II.1 Effect of pixilation on growth variables over time, means and CV over simulations

The ten stands of each scenario grew during 200 years without thinning. The model provided the following stand-level variables annually:

- Total number of surviving trees N.
- Dominant diameter Ddom and mean diameter Dg (respectively root mean square of the diameters of the 100 largest trees in the stand per hectare and root mean square of the diameters over the entire stand). Note: a distinction must be made between the Ddom of the pixels and that of the stand. In the figures, Ddom is computed at the stand level. It is not an average of the pixel Ddom.
- Dominant height Hdom and average height Hg (respectively root mean square of the heights of the 100 tallest trees in the stand per hectare and root mean square of the heights over the entire stand). Note: Hdom is computed at the stand level. It is not an average of the Hdom of the pixels.
- Basal area G (sum of tree sections at 1m30).

We computed the inter-simulation means and coefficients of variation (CV = inter-simulation standard deviation/inter-simulation mean*100) to compare modalities and scenarios as follows:

1. for each pixel size, we compared the effect of the three modalities - spatial positions, initial diameters and initial number of trees - on the inter-simulation means and CVs of the variables;
2. for each modality, we compared the effect of pixel sizes on the inter-simulation means and CVs of the variables; we examined the differences between the inter-simulation means of the two smaller pixel sizes and the 60mx60m reference for each variable after 100 years.

### II.2 Effect of pixilation on within-stand variation of growth variables

We studied the effect of pixilation on the predicted heterogeneity of tree density, basal area and mean diameter by comparing the within-stand variation of variables over three conditions (Figure 2):

- a stand grown as a single growth unit 60x60m;
- a stand grown as a single growth unit 60mx60m, then the variables were first computed on a 15mx15m pixel grid before aggregation at stand level;
- a stand grown on 15mx15m independent pixels.

Here, we replicated each comparison in two cases with different spatial positions of the trees.


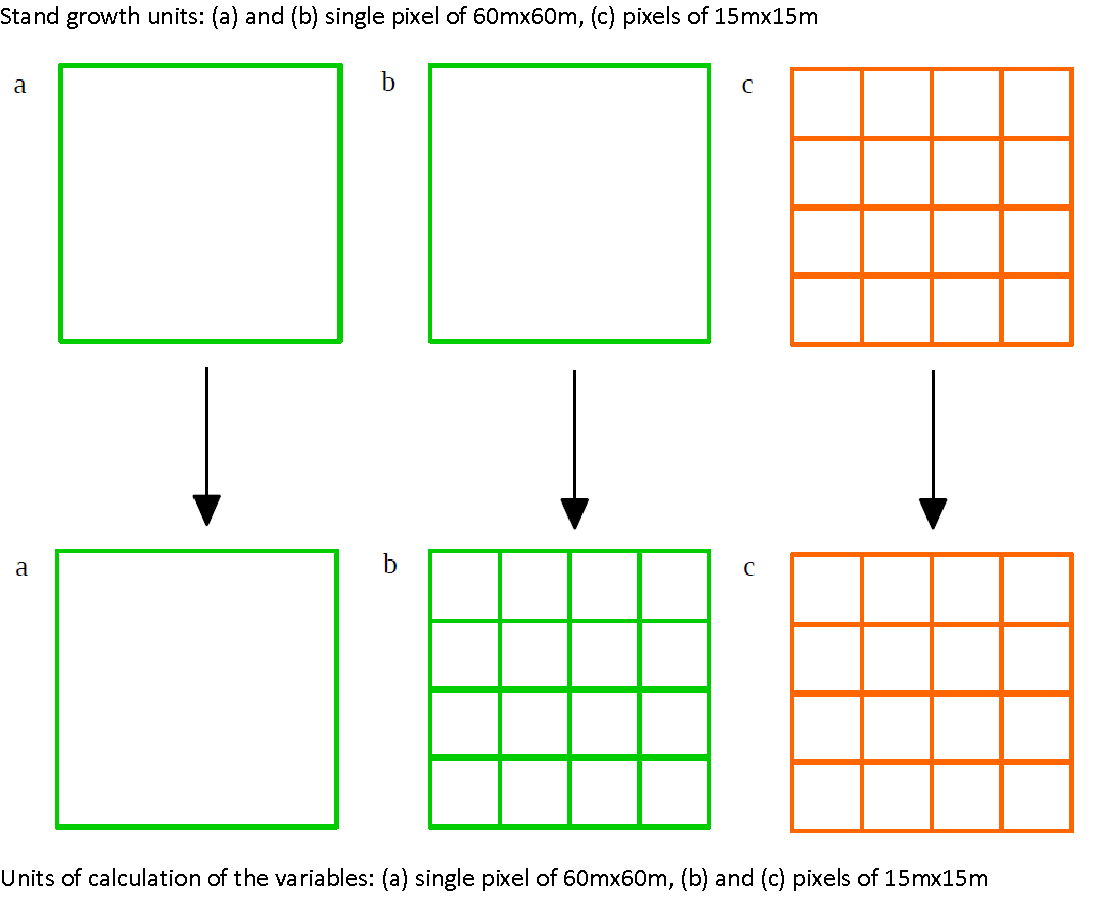


*Figure 2: Diagram of the three scenarios to test the effect of pixilation on spatial heterogeneity*

## III. Results

### III.1. Effect of pixel sizes and inventory composition on inter-simulation means and coefficients of variation

**a. Density N** (Figure 3)

Decreasing pixel size decreases the final predicted density compared to the reference. At year 100, the average number of trees of the simulations with 15mx15m and 10mX10m pixels are respectively 12.90 and 23.40 individuals less than the reference numbers 116.5 and 119 (Table 1).

As expected, variation in initial densities between inventories induces inter-simulation variation in densities (Figure 3).

The first mortalities occur between years 2 and 20. The self-thinning threshold varies with the average tree circumference of the pixel. In a multi-pixel stand, the fluctuation of densities between pixels leads to a spreading out of mortality over time, in contrast to a single-pixel stand where the first mortality occurs later and is more abrupt (Figure 3 g to i). The more pixels there are, the more the spatial positions, initial diameters and initial number of trees induce fluctuations in the average circumference between simulations of each pixel and thus the number of dead trees between simulations (Figure 3 j to l).

For the 60mx60m reference, the growth model does not depend on the spatial position of trees. The average growth is therefore the same in all 10 simulations and mortality is identical between simulations, and the CV over spatial positions is therefore null (Figure 3 d and j). With pixilation, different spatial tree positions in the stand induce differences in tree densities and diameters at pixel-level, which induces a variation of stand dynamics across simulations.

Spatial positions increase inter-simulation fluctuations less than initial diameters and density. The lower the initial diameter, the later the mortality occurs. The variation of the initial densities thus leads to a strong inter-simulation variation of the average diameters. This is why, regardless of the pixel size, initial densities increase CVs the most (Figure 3 d to f).

*Table 1: Average bias of predicted density compared to the reference in number of trees (in %) at year 100 for pixel sizes 15mx15m and 10mx10m and for each modality*

|  | Spatial position | Initial diameters | Initial densities |
| --- | --- | --- | --- |
| 15mx15m | -12.20 (-10.25%) | -12.40 (-10.38%) | -12.90 (-11.07%) |
| 10mx10m | -23.40 (-19.66%) | -21.70 (-18.16%) | -20.90 (-17.94%) |


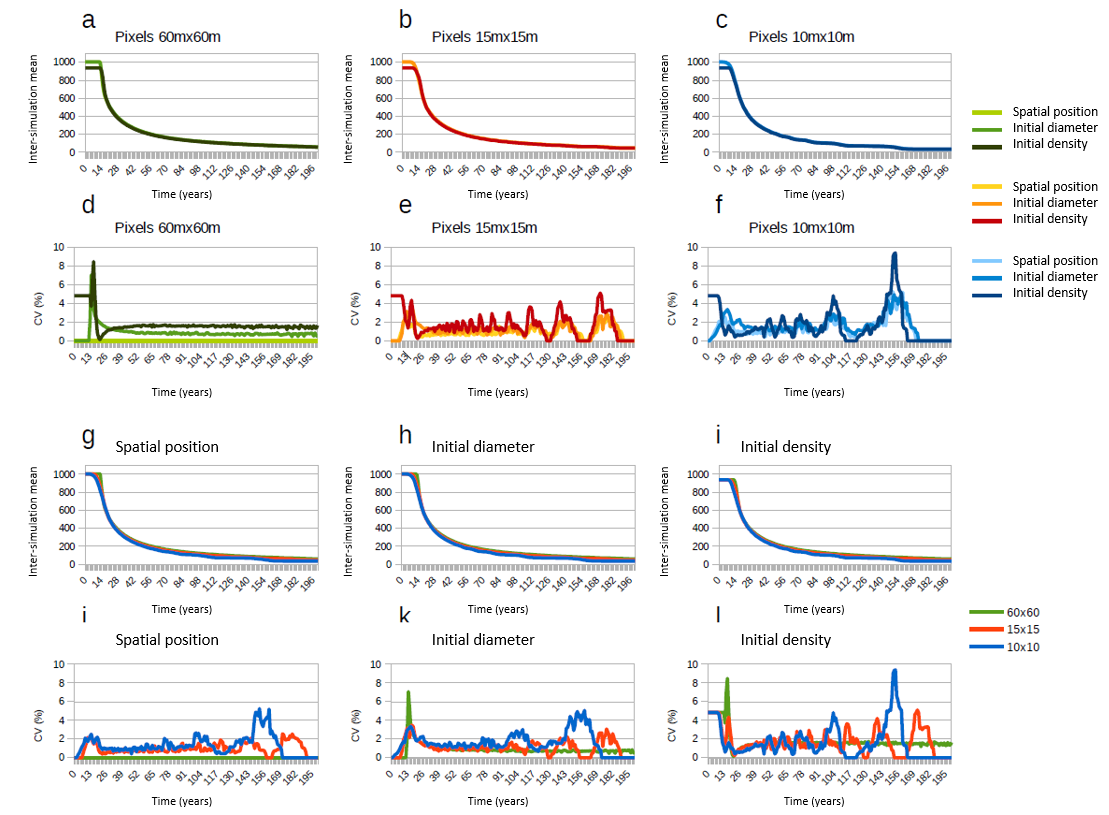


*Figure 3: Comparison of inter-simulation means and CVs of the evolution of population density (number of surviving trees) between scenarios. a to c: comparison of inter-simulation means among modalities for each pixel size. d to f: comparison of CVs among modalities for each pixel size. g to i: comparison of inter-simulation means among pixel sizes for each modality. j to l: comparison of CVs among pixel sizes for each modality.*

**b. Dominant diameter Ddom** (Figure 4)

As the size of the growth units decreases, the predicted Ddom is greater than the reference (Figure 4 g to i). At year 100, the average Ddom of simulations with 15mx15m and 10mX10m pixels are at most 2.56 and 4.82 cm above the reference, respectively (Table 2).

Regardless of pixel size, the CV of spatial positions is lower than the CV of initial diameters, which is lower than the CV of initial densities (Figure 4 d to f). The smaller tree density in the inventory, the later mortality will occur. The variation in initial densities thus leads to a strong inter-simulation variation in mean diameters, which results in a higher CV for simulations with variations in initial densities.

As for the density, null CVs are expected in the reference case of a single growth unit when spatial position varies because the original growth model is independent from the spatial position of trees, and thus the dominant diameter is independent of the spatial position of the trees (Figure 4 d and j).

*Table 2: Average bias of predicted Ddom compared to the reference in cm (in %)) at year 100 for pixel sizes 15mx15m and 10mx10m and for each modality*

|  | Spatial position | Initial diameters | Initial densities |
| --- | --- | --- | --- |
| 15mx15m | +2.51 (+4.15%) | +2.56 (+4.24%) | +2.34 (+3.82%) |
| 10mx10m | +4.57 (+7.56%) | +4.82 (+7.99%) | +4.67 (+7.62%) |


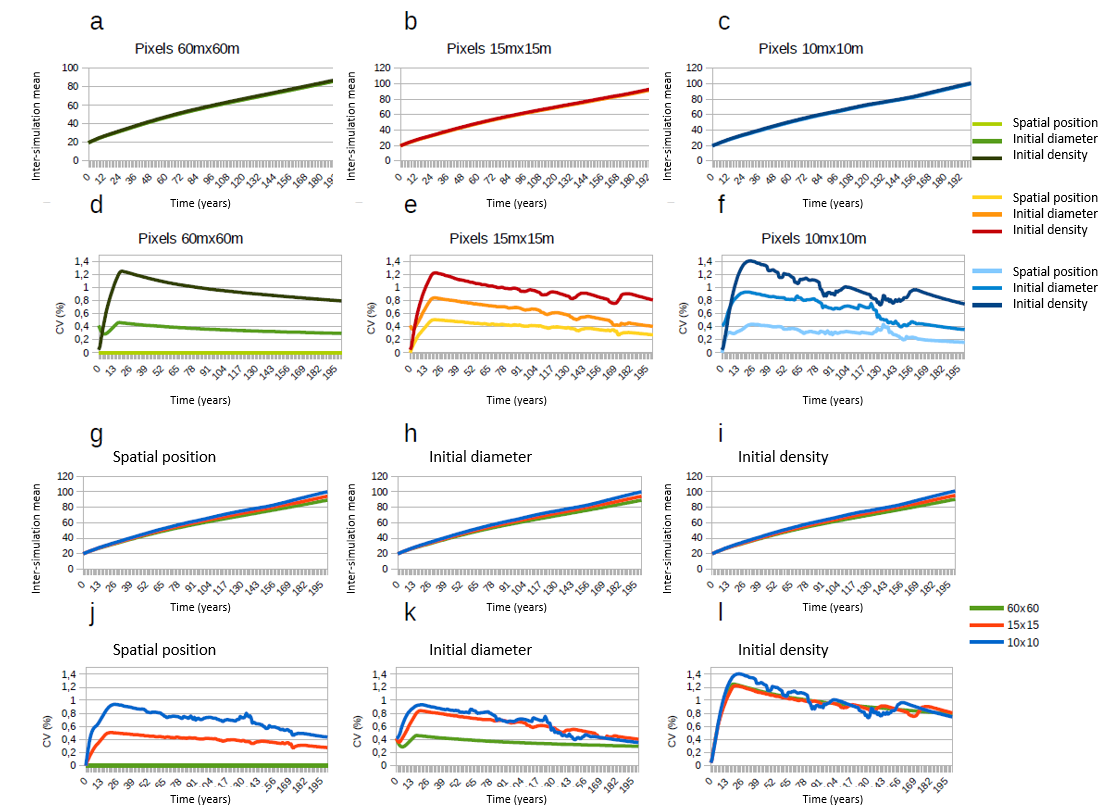


*Figure 4: Comparison of inter-simulation means and CVs of dominant diameter Ddom between scenarios. a to c: comparison of inter-simulation means among modalities for each pixel size. d to f: comparison of CVs among modalities for each pixel size. g to i: comparison of inter-simulation means among pixel sizes for each modality. j to l: comparison of CVs among pixel sizes for each modality.*

With pixilation, as the size of the growth units decreases, the coordinates of the trees change the initial tree density and initial distribution of tree diameters, thus affecting the parameters of the growth model at pixel level. The CV therefore increases with decreasing pixel sizes (Figure 4 j). However, it can be seen in Figure 4 k and l that after about 120 and 80 years, respectively, the CV of the simulations with 10mx10m pixels is less than or equal to that of the simulations with 15mx15m pixels. At the beginning of the simulations, variations in initial diameters or initial numbers cause some heterogeneity in the growth model parameters across pixels. The more pixels there are, the greater this heterogeneity becomes. Over time, mortality homogenizes the tree densities per pixel and decreases the differences in Ddom CVs.

**c. Average diameter Dg** (Figure 5)

As the size of the growth units decreases, the Dg becomes larger than the reference (Figure 5 g to i). At year 100, the mean Dg of the simulations with 15mx15m and 10mX10m pixels are at most 1.80 and 3.22 cm higher than the reference mean Dg, respectively (Table 3). Inter-inventory variation in initial diameters causes inter-simulation variation (CVs of about 1.5% at the start of simulations, Figure 5k).

As with Ddom, the CVs for spatial positions scenarios in the reference case of single 60mx60m growth unit are null because within a pixel all individuals have the same growth model parameters (Figure 5 d and j).

In all other scenarios, there is a peak in CV between 15 and 20 years. This peak is due to early self-thinning mortality that removes more or fewer trees depending on the simulation. The self-thinning threshold varies with the average tree circumference of the pixel. The variation of initial spatial positions, diameters and densities induces a variation of the average circumference per pixel. In a stand spread over several pixels, the fluctuation of densities between pixels leads to a spread in time of the mortality, in contrast to a stand on a single pixel where the first mortality will be more brutal. The more pixels there are, the more gradually the self-thinning threshold is reached and the less important the CVs are between 15 and 20 years (Figure 5 j to l).

After 20 years, CVs converge as mortality homogenizes the density of trees per pixel and thus diameter growth (Figure 5 j to l).

Spatial positions increase inter-simulation fluctuations less than initial diameters and density. The lower the initial density, the later mortality will occur. The variation of the initial densities thus leads to a strong inter-simulation variation of the average diameters. This is why, whatever the pixel size, the initial densities increase the CVs of Dg the most (Figure 5 d to f).

*Table 3: Average bias of predicted Dg compared to the reference in cm (in %) at year 100 for pixel sizes 15mx15m and 10mx10m and for each modality*

|  | Spatial position | Initial diameters | Initial densities |
| --- | --- | --- | --- |
| 15mx15m | +1.78 (+3.02%) | +1.79 (+3.04%) | +1.80 (+3.01%) |
| 10mx10m | +3.22 (+5.46%) | +3.22 (+5.46%) | +3.09 (+5.16%) |


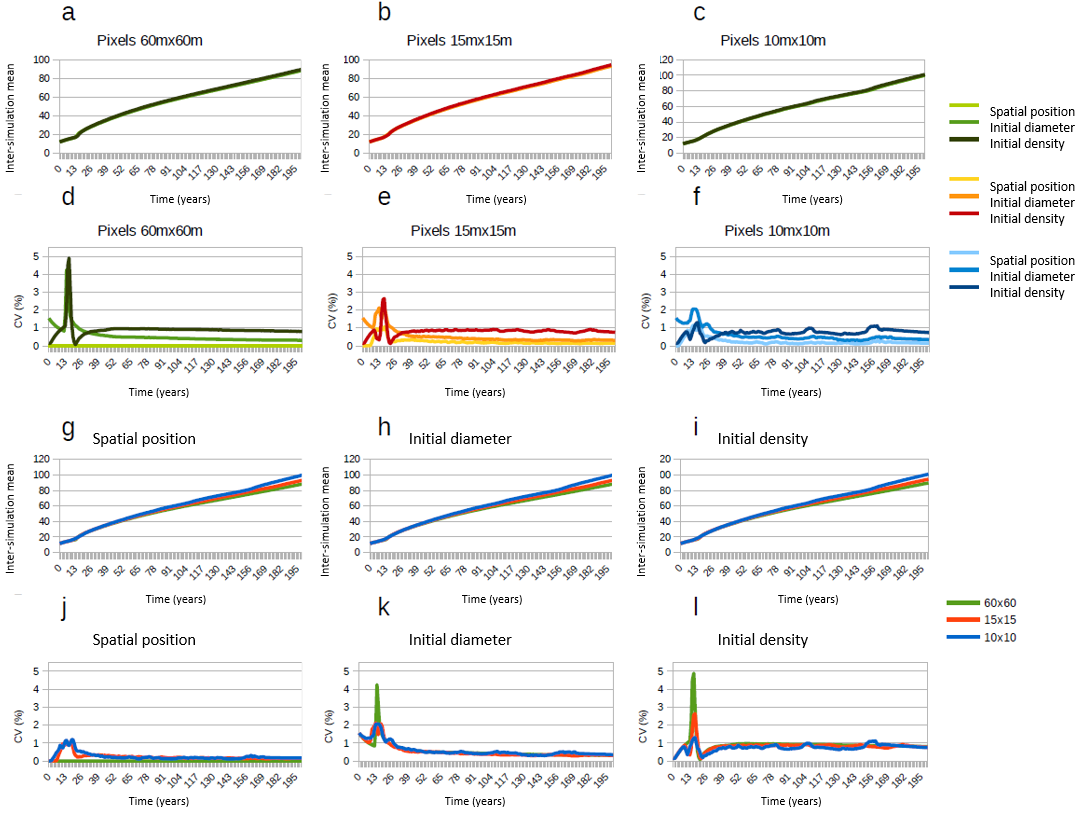


*Figure 5: Comparison of inter-simulation means and CVs of mean diameter between scenarios. a to c: comparison of inter-simulation means among modalities for each pixel size. d to f: comparison of CVs among modalities for each pixel size. g to i: comparison of inter-simulation means among pixel sizes for each modality. j to l: comparison of CVs among pixel sizes for each modality.*

**d. Dominant height Hdom** (results not shown)

The decrease in pixel size implies a higher predicted Hdom than the reference. However, the average Hdom of the simulations with 15mx15m and 10mx10m pixels are very close. For example, at year 100, the average Hdom of the simulations with 15mx15m and 10mX10m pixels are at most 1.03 and 1.11 m higher than the reference, respectively (Table 4). The bias induced by a decrease in unit growth is therefore not proportional to pixel size.

Tree height growth varies with individual diameter, Ddom and Hdom of the pixel. The variation in tree position within the single pixel reference does not affect Ddom and Hdom. As with Ddom, in the reference case for the spatial position modality, the CVs are therefore null.

Decreasing tree density across inventories causes inter-simulation fluctuation in Ddom of pixels where individuals have disappeared. In contrast, when initial diameters vary between inventories, the Ddom of all pixels vary between simulations. The CV of simulations with varying initial diameters is therefore higher than that of simulations with varying initial densities.

The CV of simulations with 10mx10m pixels (area = 0.01 ha) is null because we reach the limits of the model. Indeed, the Ddom is computed on the 100 largest trees per hectare but on a pixel of 0.01 ha with very few trees, the Ddom is the diameter of the largest tree of the pixel. When we then calculate the height of this tree with the relation:

h=$1.3+\frac{Hdom-1.3}{1-exp(-b_{2}*Ddom*\pi)}$*1-exp(-$b_{2}$*diameter*π)

with $b_{2}$=1.73 x 10-2 ${cm}^{-1}$

As diameter = Ddom, the previous equation becomes h = Hdom of the pixel. In each pixel, regardless of the diameter of the largest tree, we therefore have a tree whose height is Hdom. Moreover, since Ddom is equal to the maximum diameter, the maximum height cannot exceed Hdom. In each pixel, the tallest tree therefore has a height equal to Hdom. When we calculate the dominant height at the stand level on the 36 tallest trees (0.36 ha * 100), we average the tallest tree of the 36 pixels i.e. Hdom. There is therefore no inter-simulation variation in Hdom for the 10mx10m pixels.

For simulations with 15mx15m pixels, the CV is very low (0.076% maximum). The area of the pixels is 0.225 ha so we should calculate Ddom on two individuals but if the number of trees is less than ten, then Ddom is defined as the diameter of the largest tree in the pixel. As for the 10mx10m pixels, the tallest tree in each pixel has a height equal to Hdom and all other trees are strictly less than this height. For the calculation of Hdom, 16 trees out of 36 therefore have an identical height within and between simulations, which explains the low inter-simulation variation.

*Table 4: Average bias of predicted Hdom compared to the reference in m (in %) at year 100 for pixel sizes 15mx15m and 10mx10m and for each modality*

|  | Spatial position | Initial diameters | Initial densities |
| --- | --- | --- | --- |
| 15mx15m | +1.03 (+2.51%) | +1.03 (+2.51%) | +1.03 (+2.51%) |
| 10mx10m | +1.05 (+2.56%) | +1.05 (+2.56%) | +1.11 (+2.71%) |

**e. Average height Hg** (results not shown)

As with Hdom, decreasing pixel size causes higher predicted Hg than the reference. This overestimation varies very little with the pixel sizes tested. At year 100, the average Hg of the simulations with 15mx15m and 10mX10m pixels are at most 1.05 and 1.09 m higher than the reference, respectively (Table 5).

As with Hdom, the CVs are null for the 60mx60m reference - spatial positions scenario. Between 15 and 20 years, there is a peak in CVs for all other scenarios. As for Dg, this peak is due to mortality.

Before 75 years, the CV of the simulations with varying initial diameters is slightly higher than that of the simulations with varying initial densities for all pixel sizes for the same reason as Hdom: varying initial diameters fluctuates the Ddom of all pixels while varying initial densities fluctuates the Ddom only of the pixels where a tree has been removed.

After 75 years, the CVs of the three modalities tend to zero because mortality has homogenized the mean circumferences per pixel and thus indirectly the Ddom, Hdom, and growth model parameters. Thus, height growth varied little between simulations.

*Table 5: Average bias of predicted Hg compared to the reference in m (in %) at year 100 for pixel sizes 15mx15m and 10mx10m and for each modality*

|  | Spatial position | Initial diameters | Initial densities |
| --- | --- | --- | --- |
| 15mx15m | +1.05 (+2.57%) | +1.04 (+2.54%) | +0.98 (+2.4%) |
| 10mx10m | +1.09 (+2.67%) | +1.09 (+2.67%) | +1.03 (+2.52%) |

**f. Basal area G** (Figure 6)

As pixel size decreases, the predicted stand basal area is lower than the reference. Decreasing pixel sizes causes temporal fluctuations in the inter-simulation mean basal area. The smaller the pixel size, the larger the amplitude of the fluctuations (Figure 6 g to i). These fluctuations are due to mortality events. The older the stand, the larger the diameter of the trees that are removed by self-thinning. Fluctuations are amplified over time by squared diameter (diameter^2^). Fluctuations in the inter-simulation means of basal area over time also vary with the modality tested. However, there does not appear to be a pattern (Figure 6 a-c). At year 100, the mean Gs of the simulations with 15mx15m and 10mX10m pixels are underestimated by up to 1.85 m² and 3.47 m² respectively (Table 6).

For the reference case of single growth unit 60mx60m with variation in spatial positions, the CV is null because the parameters of the growth model are identical between simulations (Figure 6 d and j).

The CVs are correlated with the CVs of the total number of trees N. As with the latter, the CV of simulations with 10mx10m pixels is higher than that of simulations with 15mx15m pixels (Figures 3 d to l and 6 d to l) because the more pixels there are, the more the mortality of each pixel varies between simulations.

Spatial positions increase inter-simulation fluctuations less than diameters and initial densities. The smaller the initial density, the later the mortality will occur. The variation of the initial density thus involves a strong inter-simulation variation of the diameters. This is why, regardless of the size of the pixels, the initial density increases the CVs the most (Figure 6 d to f).

*Table 6: Average bias of predicted G compared to the reference in m² (in %) at year 100 for pixel sizes 15mx15m and 10mx10m and for each modality*

|  | Spatial position | Initial diameters | Initial densities |
| --- | --- | --- | --- |
| 15mx15m | -1.55 (-4.76%) | -1.59 (-4.87%) | -1.85 (-5.65%) |
| 10mx10m | -3.47 (-10.66%) | -3.15 (-9.66%) | -3.03 (-9.25%) |


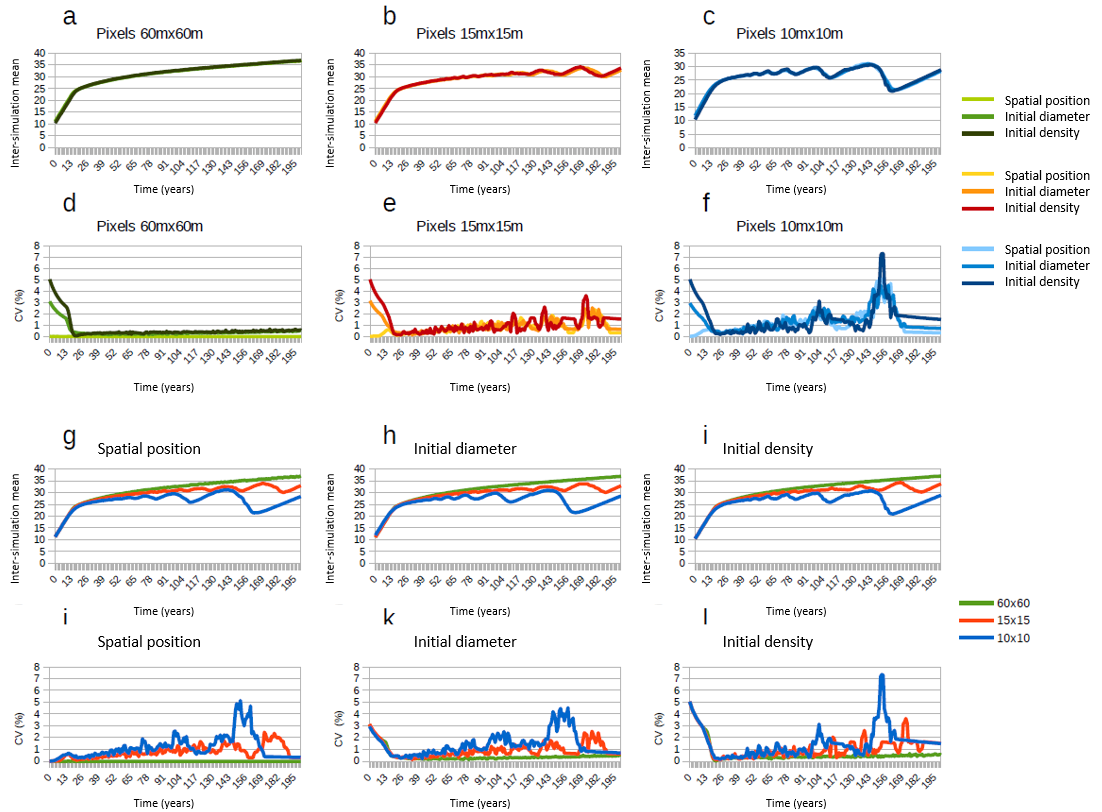


*Figure 6: Comparison of inter-simulation means and CVs of basal area among scenarios. a to c: comparison of inter-simulation means among modalities for each pixel size. d to f: comparison of CVs among modalities for each pixel size. g to i: comparison of inter-simulation among pixel sizes for each modality. g to i: comparison of inter-simulation means among pixel sizes for each modality. j to l: comparison of CVs among pixel sizes for each modality.*

### III.2. Effect of pixilation on spatial heterogeneity of variables

When the stands have grown in exactly the same way, with a single growth unit of 60mx60m, computing variables on the whole growth unit or on a 15mx15m pixel grid does not change the tree density per ha (Nha) and basal area per ha (Gha) at year 100. However, the Dgs differ slightly (from -0.19% to +0.07%) because they are not exactly computed on the same trees: the Dg for the single growth unit is computed on the 100 largest trees in the stand while the Dg for a 15mx15m pixel grid is an average of the largest tree in each pixel.

With independent pixel growth, the homogenized mortality across pixels decreases the predicted number of trees in the whole stand as explained above. The decrease in tree density allows a faster growth of trees on independent pixels. Since the first mortality occurs between years 2 and 20, the mortality at year 100 has already decreased the density of trees in the case of pixilation compared to the reference case of a single growth unit. The predicted Nha and Gha (respectively Dg) with independent pixel growth are therefore lower (respectively higher) than those for a single growth unit.

As expected, independent pixel growth increases coefficients of variation in Dg relative to a single growth unit (2.7 to 3.2 times). However, the coefficients of variation with independent pixel growth remain below 3%. Thus, pixilation slightly increases spatial heterogeneity. In contrast, for Nha and Gha the coefficients of variation are lower with independent pixel growth (-3.8 to -5.9 times and -6.6 to -10.4 times the Dg with a single growth unit, respectively). Pixilation homogenizes the number of individuals between pixels (with independent pixel growth the densities at year 100 vary between 6 and 7 trees per pixel while with a single growth unitthe densities can vary from 1 to 12) which decreases the spatial heterogeneity of variables between pixels. The spatial heterogeneity produced by pixilation is therefore negligible compared to the homogenizing effect of mortality (Table 7).

*Table 7: Means, standard deviations and coefficients of variation (CV) of total number of trees per hectare, basal area per hectare and mean diameter in three scenarios: a stand grown on a single 60mx60m growth unit; a stand grown on a single growth unit 60mx60m and then where variables computed on a 15mx15m pixel grid; and a stand grown on independent 15mx15m pixels. The minimum and maximum numbers of trees per pixel are shown for each scenario.*


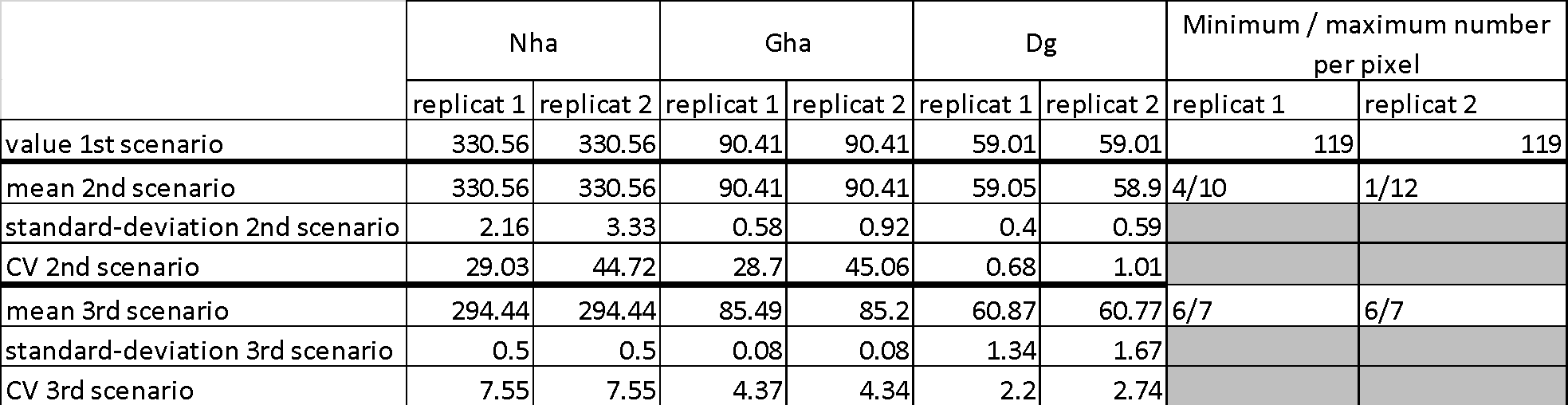


In summary, mortality rapidly homogenizes the number of trees between pixels. Pixels grow independently, but in a very similar manner. Therefore, pixilation decreases spatial heterogeneity in total number of trees and basal area and very slightly increases spatial heterogeneity in tree diameters.

## IV. Conclusion

For pixel sizes 15mx15m and 10mx10m, the limits of the model are reached and this affects the temporal evolution of the dominant diameters and heights. These limits must be kept in mind when interpreting the simulations.

We observed that the first mortalities lead to an increase in CVs between 15 and 20 years in diameter and mean height. Beyond that, mortality homogenizes tree densities among pixels and among simulations. Users may be advised to run simulations for more than 30 years in order to reduce variations between simulations. However, the longer the simulations, the greater the bias due to decreasing growth unit sizes.

Initial spatial positions, diameters, and number of trees have little effect on the inter-simulation means. In contrast, CVs vary differently by modality. Spatial positions are the modality that increases CVs the least. Most of the time, it is the variations in initial densities between inventories that increase CVs the most (except for heights, but CVs are very largely biased by the limits of the model). When interpreting the simulations, the effects of inventory composition on the results should be considered, especially if initial densities vary.

For all measured variables, the inter-simulation means of the simulations with 15mx15m pixels are closest to the inter-simulation means of the predictions given by the reference simulations. Depending on the variable, decreasing growth unit sizes result in a positive or negative bias with respect to the reference size. CVs for simulations with 15mx15m pixels are often smaller than those for 10mx10m pixels (Table 8).

Pixilation slightly increases spatial heterogeneity of mean diameters and homogenizes total density and basal area.

*Table 8: Selection of the most appropriate pixel size after reminding the pixel size with the least deviations from the reference inter-simulation means and the lowest and the lowest CVs, then reminding the maximum average bias among the 3 modalities at year 100 for the selected pixel size*

| Variable | Pixel size with the least deviations from the reference inter-simulation means | Size of pixels with the lowest CVs | Selected pixel size | Mean bias for the selected pixel size |
| --- | --- | --- | --- | --- |
| N | 15mx15m | 15mx15m | 15mx15m | -12.90 trees (-11.07%) |
| Ddom | 15mx15m | 15mx15m before 80-120 years and then 10mx10m | 15mx15m | 2.56 cm (+4.24%) |
| Dg | 15mx15m | 15mx15m ≈ 10mx10m | 15mx15m | 1.80 cm (+3.01%) |
| Hdom | 15mx15m | wrong CV | 15mx15m | 1.03 m (+2.51%) |
| Hg | 15mx15m | wrong CV | 15mx15m | 1.05 m (+2.57%) |
| G | 15mx15m | 15mx15m | 15mx15m | -1.55 to -1.85 m^2^ (-4.76 to -5.65%) |

## V. References

Courbet, F. (2002). Modélisation de la croissance et de la qualité du Cèdre : intégration des modèles dans Capsis. In : Rapport convention MAPA-DERF / INRA n° 61.45.47/01. <https://hal.archives-ouvertes.fr/hal-03654591>

Sambakhe, D., Fortin, M., Renaud, J.P., Deleuze, C., Dreyfus, D., Picard, N. (2014). Prediction bias induced by plot size in forest growth models. *Forest Sciences*, 60, 1050-1059. http://dx.doi.org/10.5849/forsci.13-070

# B- Consequences on the regeneration process

When regeneration occurs, each concerned pixel receives a number of seedlings determined by the contribution of all mother-trees given their female fecundity and their distance to the center of the pixel following a dispersal kernel. The seedlings are initialized with a DBH drawn from the expected distribution of DBH given tree density at recruitment age. The stand dynamic processes in the new cohort only start when it reaches the recruitment age.

At the year of recruitment age, dynamic processes start with a first step of growth followed by self-thinning. This first self-thinning acts as a “demographic regulation step” that immediately reduces seedling density to the “carrying capacity”, i.e. the maximum density allowed given the root mean square of circumferences in the pixel as defined in the self-thinning model, if necessary. Indeed, the initial number of seedlings created in the pixel does not result from a previous forest dynamics process but from seed dispersal and it may be much higher than the carrying capacity. This demographic regulation step uses the self-thinning process, i.e. the smallest trees are removed. In the following years after this step, tree densities are determined by the forest dynamics process and the decrease in tree density by self-thinning is more progressive.

The critical point here is the initial number of seedlings created in a pixel which has three main consequences: (i) it determines the intensity of the demographic regulation step, (ii) it determines the probability of extreme genotypic values, (iii) it determines the expected distribution of DBH for seedling initialization. Here, we further examine how these consequences could theoretically affect the evolutionary process after regeneration.

## I. The number of seedlings is arbitrarily scaled and depends on pixel size

In Luberon2, we use a female fecundity function relating the number of seeds produced (number of cones and number of seeds per cone) to tree dimension and a seed dispersal function, which were both calibrated on empirical data for *Cedrus*. These functions provide the appropriate relative contributions of the different mother trees to the seed rain in each pixel, but using the total number of seeds received in all pixel areas might rapidly exceed the computational limits that we allocate to the model (Luberon2 can handle tens or even hundreds of thousands of trees for decades but handling tens of millions of seeds would be another issue). In addition, in a classical real life situation, the huge majority of seeds does not reach the recruitment stage and does not contribute to the post-recruitment forest dynamic processes that the model accounts for. In the current version of the model, we get the relative contribution of the different mother trees to the seed rain at the center of the pixel from the calibrated fecundity and seed dispersal functions, but we arbitrarily scaled the expected number of seedlings remaining until recruitment as equal to the content of 1m² of the seed rain.

With the default pixel size (15mx15m), considering a classical stand structure before regeneration, this scaling factor generally leads to an initial seedling density around or above the carrying capacity, which is immediately corrected by the demographic regulation step mentioned above at recruitment age. In some cases, if the seed trees are small or very few, the initial seedling density obtained in a pixel can be below the carrying capacity: in this case, the demographic regulation step does not remove any tree. The question of the possible evolutionary consequences of the intensity of the demographic regulation step is treated below.

With larger pixels sizes, the number of seedlings rapidly drops for two reasons: (i) at the whole scene level, larger also means fewer pixels and the number of seedlings remains computed on the same basis of 1m² seed rain per pixel, (ii) larger pixels increase the distances between each seed-tree and each pixel center, which rapidly has a severe impact on the number of dispersed seeds due to the dispersal function. Thus, it is important to consider that using pixels size larger than the default value can rapidly provide very low seedling densities, which may become an issue depending on the objectives of the simulations.

## II. The demographic regulation step has no short-term impact on selection for growth vigor

The initial number of seedlings has an arbitrary scale, therefore the intensity of the demographic regulation step, when all seedlings above the carrying capacity are removed in one step, is also arbitrary. The demographic regulation uses the self-thinning process that removes trees from the smallest DBH value upwards. The initial DBH values of the created seedlings are taken at random in a distribution, independently of their genotype for growth vigor QTL. So, at initialization, seedling DBH and genotypes are completely uncorrelated. Due to the sequence of processes in the model, the demographic regulation step proceeds after a first step of growth where growth vigor genotype starts to act on the DBH. However, we checked that at this very first step in the seedlings dynamics, there is still no correlation between individual DBH and genotypes: depending on the site index and initial tree size, the correlation only starts to appear two to four years after recruitment. Therefore, the demographic regulation step has no immediate selective impact on vigor: having many seedlings in excess, and thus intensive demographic regulation, does not represent a hidden selection on growth vigor.

## III. The initial number of seedlings has a small impact on long-term evolvability of all traits

Creating many seedlings in excess can have a long-term evolutionary impact because it determines the total number of recombined QTL genotypes in the initial population, possibly increasing the probability of rare extreme genotype values. The range of genotypic values is not affected by the genetically random demographic regulation step but it will feed subsequent self-thinning selection, allowing for higher response to selection when the cohort gets older. In other words, the initial number of seedlings before demographic regulation represents the amount of recombination offered to selection. We checked that this effect may happen but its quantitative impact on the overall evolutionary trajectory is negligible compared to the effect of different self-thinning intensities occurring after recruitment.

## IV. The number of seedlings has a tiny impact on the kinetics of selection for growth vigor

Creating many seedlings in a pixel also affects the distribution of their initial DBH values because this distribution is adjusted to the density: the more numerous seedlings, the smaller their initial DBH values. We previously mentioned that the initial DBH are uncorrelated with the growth vigor genotypic values and that this correlation progressively establishes during tree growth. In theory, if the initial DBH of seedlings is smaller, the imprint of growth vigor genotype on DBH, and therefore the selection pressure on vigor, arrives sooner. In practice, this is negligible and, as previously mentioned, the correlation between DBH and growth vigor genotype already starts after two to four years even in the case of “big” seedlings.

## V. Conclusion

In this individual-based demo-genetic model, an initial number of seedlings, not seeds, is created in each pixel and it is regulated according to the carrying capacity at the recruitment age. Such demographic regulation step is a classical feature in demo-genetic models but the particularity here is that the initial number is arbitrarily scaled based on the expected number of seeds. Therefore, two pixels with different expected seed rain (quantitatively different) will receive different number of seedlings, accordingly, but the choice of the scaling factor between the expected number of seeds and the number of seedlings may end with different situations relatively to the carrying capacity: the number of seedlings can be higher than the carrying capacity, in which case demographic regulation operates, or lower, with no regulation in this case.

We examined the various possible consequences of the initial number of seedlings, i.e. the consequences of the scaling option chosen to transform the seed rain into a number of seedlings, on the evolutionary trajectory. We conclude that within a single simulation scenario, and using the default pixel size, the number of seedlings at recruitment age after regulation globally falls in the magnitude of what is observed empirically in reality. We also conclude that initializing different numbers of excessive seedlings above the carrying capacity has negligible consequences compared to the main evolutionary drivers operating in the system.

However, we identify two critical situations where the number of seedlings resulting from the current scaling option may become critical. A first situation is when larger pixels are used, which can drastically decrease the amount of seedlings at the whole scene level, down to very unrealistic numbers for which genetic drift would probably emerge as a main evolutionary driver. A second situation is when different scenarios are compared in which regeneration occurs with very different sizes or numbers of seed-trees. In this case, very different initial seedling numbers can result in very different importance given to the side-effects of the demographic regulation step described above: even though the side-effects are limited, could these differences become critical for the comparison of interest for the user? For both situations, we recommend making preliminary simulations and deciding if there is a need to change the scaling option that determines the initial number of seedlings. If needed, there are at least three options to do this: (i) a simple option is to adjust the number of seeds per cone in the corresponding species file, (ii) a second option is to add specific ad-hoc interventions at recruitment age, acting as “manual” demographic regulation for each scenario whenever needed, (iii) a third option for developers is to change the area of the seed rain used to scale the number of seedling within the code of the model. Note that options (i) and (iii) will make the same changes in all pixels of all scenarios of the same project, keeping their differences in another scale (e.g. using these options to force all pixels to receive more seedlings than the carrying capacity will also increase the differences of demographic regulation intensity), while option (ii) allows differential adjustment to homogenize the initial number of seedlings among pixels or scenarios.
