# Supplementary material for: A demo‐genetic model shows how silviculture reduces natural density‐dependent selection in tree populations
Source: Evol Appl. 2023 Oct 13;16(11):1830–44. doi: 10.1111/eva.13606 (PMC10681482; doi:10.1111/eva.13606)
Supplement: Supplementary file 3 — Appendix S3 [file EVA-16-1830-s005.docx]

Appendix 3 - Growth and fecundity functions in Luberon2.

# Individual tree growth

Luberon2 relies on the original growth model calibrated for *Cedrus atlantica* (Courbet, 2002). The basic principle is that current local stand characteristics determine the conditions of individual trees’ growth, which in turn determines the future local stand characteristics for the next step. Individual tree growth follows a segmented model where annual increment in basal area is null for trees below a threshold circumference value, and increases linearly with the circumference above this threshold (Figure 1).


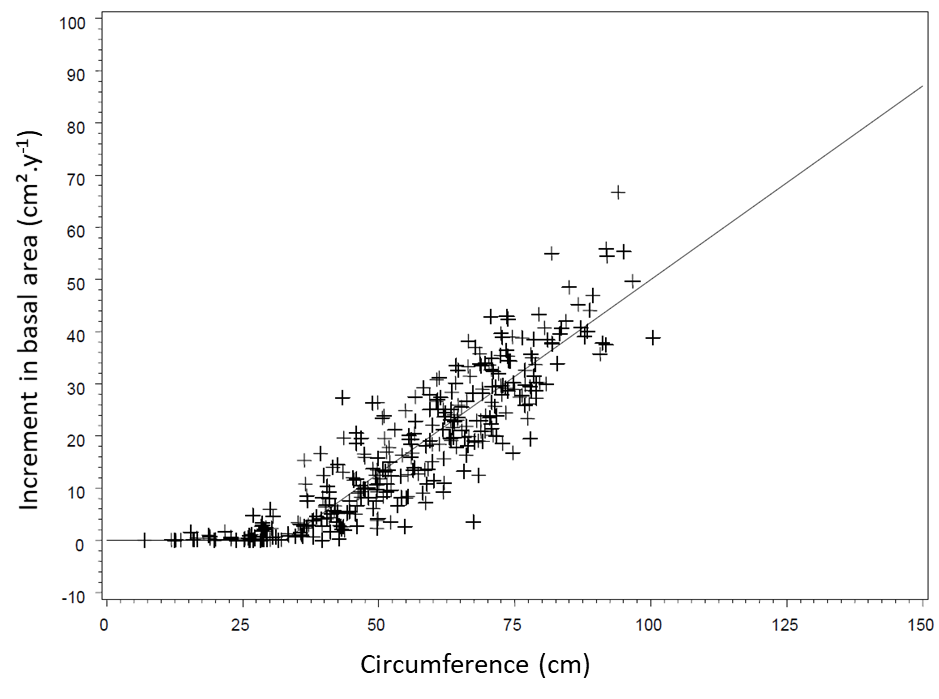


***Figure 1****: Individual annual increment in basal area as a function of tree circumference in C. atlantica: empirical individual data and model prediction (taken from Courbet, 2002).*

In Luberon2, this segmented relationship is modeled using the nonlinear function proposed by Deleuze et al (2004) with three parameters: the threshold value, $A$, the slope of the relation between annual growth in basal area and circumference, $P$, and a flexibility parameter smoothing the break around the threshold value, $m$ ($m$=1 for *C. atlantica*), resulting in the following equation of growth in basal area (above the threshold):

$${Ig}_{i}=0.5P\left( C_{i}-mA+\sqrt{\left( mA+C_{i} \right)^{2}-4AC_{i}} \right)$$

where ${Ig}_{i}$ is the increment in basal area of individual *i*, and $C_{i}$ the initial circumference. These parameters evolve during stand development, they are dynamically computed for each even-aged growth unit depending on age, stand density and site fertility, as well as species-specific constant values provided as input to the model.

Then, individual phenotypic variation in vigor is added to the diameter growth ${dD}_{i}$ as follows:

$${dD}_{i}=\frac{1}{\pi}\left( \sqrt{C_{i}^{2}+4\pi{Ig}_{i}}-C_{i} \right)+\left( Mean\_vigor+{Phenotype\_vigor}_{i} \right)$$

where $Mean\_vigor$ is a constant value common to all individuals and ${Phenotype\_vigor}_{i}$ is the individual phenotypic deviation to this value. Then, knowing the new diameter of each individual tree, allometric relations are used to derive height growth, male and female fecundity variables as well as other values at individual level, such as crown height, or at growth unit level, such as leaf area index.

# Female and male fecundity

Trees are considered as fertile from recruitment age and above a threshold DBH value parameterized for each species, i.e. 15cm for cedar and 10cm for Douglas fir. Female fecundity is determined as an ordinal variable related to tree DBH following the model originally calibrated for cedar by Bertrand (2004). Six levels of cone production are defined (in a log-scale) and, each year, the probability to reach each level depends on the DBH, as shown on Figure 2, and crown height. Once the level of cone production is drawn, the actual number of cones produced by this tree in this year is randomly drawn within the range of the fecundity level. Thus, female fecundity has a high inter-annual (for each tree) and inter-individual (among equal-size trees) stochasticity but, currently, there is no synchrony implemented (i.e. masting). The potential number of seedlings generated by each cone produced is set to an estimate of the mean number of viable seeds per cone known for each species.

The probability of a tree $i$ to fall into fecundity level $j$ is given by the following equation:

$$P_{i}^{j}=\frac{e^{\left( m^{j}-0.0114{DBH}_{i}-0.3914{crown\_height}_{i} \right)}}{1+e^{\left( m^{j}-0.0114{DBH}_{i}-0.3914{crown\_height}_{i} \right)}}$$

where $m^{j}$ is a constant given for each fecundity level, ${DBH}_{i}$ the diameter of the tree in mm and ${crown\_height}_{i}$ the crown height in m.

Individual “male fecundity” of each fertile tree, which is used to compute the probability of being the father of a recruited seedling (see above), is a relative value simply computed as the square of its DBH (in cm), it is not an absolute value.


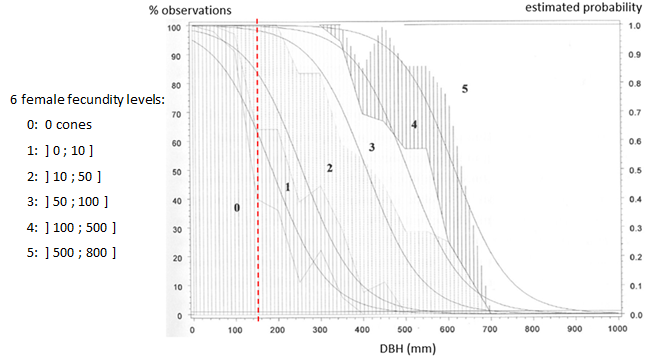


***Figure 2****: female fecundity as a function of DBH (regardless of crown height in this figure) in C. atlantica, observed data (hatching) and estimated probabilities (smooth lines) (Bertrand 2004). The red dotted line represents the sexual maturity threshold used in Luberon2, above this threshold the fecundity level is randomly drawn each year depending on tree DBH (e.g. a tree with DBH=400mm will have higher probability to fall into level 2 or 3).*

# References

Bertrand A, 2004. Dynamique naturelle du Cèdre de l'Atlas (*Cedrus atlantica* Manetti) au Mont Ventoux. Approche spatio-temporelle et éléments de modélisation. DEA Aix-Marseille. <https://capsis.cirad.fr/capsis/help_en/luberon2>

Courbet F. 2002. Modélisation de la croissance et de la qualité du Cèdre : intégration des modèles dans Capsis. In : Rapport convention MAPA-DERF / INRA n° 61.45.47/01. <https://hal.archives-ouvertes.fr/hal-03654591>

Deleuze C., Pain O., Dhôte J.-F., Hervé J.-C., 2004. A flexible radial increment model for individual trees in pure even-aged stands. *Annals of Forest Science* 61:327–335. https://doi.org/10.1051/forest:2004026
