# Supplementary material for: A demo‐genetic model shows how silviculture reduces natural density‐dependent selection in tree populations
Source: Evol Appl. 2023 Oct 13;16(11):1830–44. doi: 10.1111/eva.13606 (PMC10681482; doi:10.1111/eva.13606)
Supplement: Supplementary file 4 — Appendix S4 [file EVA-16-1830-s004.docx]

Appendix 4 - Impacts of phenotypic variation in vigor on the predicted tree growth in Luberon2.

Luberon2 builds upon a distance-independent tree growth model that provides a baseline prediction of the annual individual diameter increment of each tree depending on environmental condition (positively), initial tree diameter (positively), age (non linearly), and competition intensity (negatively).

Here, we analyze how the addition of inter-individual phenotypic variation in a growth-related trait, vigor, changes the baseline prediction of annual tree growth and tree diameter during stand development within a generation, and across generations. To summarize the general trend:

- adding individual variation in vigor does not change the predicted mean annual growth and mean diameter until selective self-thinning occurs, and it increases these predicted means when self-thinning occurs;
- adding individual variation in vigor globally increases the predicted variance of annual growth and diameter, competition without mortality increases these variances but selective mortality reduces them;
- the evolutionary rate of growth traits is higher when the environmental component of phenotypic variation has a higher proportion of fixed (vs variable) effects.

The consequences of adding individual variation in the model depend on the environmental and genetic components of the phenotypic variance and on the population dynamics, as described below.

# Different types of inter-individual variation and expected impacts on growth prediction

In the demo-genetic model Luberon2, we define a variable growth-related trait, vigor, as the deviation of the annual diameter increment of a tree to the baseline prediction by the original growth model. To account for genetic, micro-geographic and temporal variation in individual tree vigor, the predicted annual diameter growth of a tree *i* at year *t*, *dD_it_*, is obtained by adding a phenotypic value to the baseline prediction of the original growth model:

***dD_it_* = *dD_0it_* + *P_it_* = *dD_0it_* + *G_i_* + *E_Fi_* + *E_Vit_***

where *dD_0it_* is the baseline prediction of the growth model and *P_it_* is the phenotypic value of vigor. The phenotypic value of vigor has three components: the genetic value, *G_i_*, a fixed environmental individual effect, *E_Fi_*, and a variable environmental individual effect, the “inter-step” component of the environmental variance, *E_Vit_*. The genetic value of an individual is determined by its genotype; it is unique and remains constant through time. The fixed environmental individual effect accounts for tree-level micro-geographic heterogeneity that confers to an individual a growth advantage, or disadvantage, compared to the local environment shared with its neighbors, it is also a unique and constant value. The variable environmental individual effect accounts for tree-level annual stochastic events that may affect individual tree growth independently of its neighbors (beyond the local competition effect that is already integrated in the baseline prediction of the growth model), it varies annually for each individual.

Therefore, the individual tree growth model consists of four terms:

- *dD_0i_* , the deterministic baseline prediction of the growth model;
- *G_i_* , an individual-level random term, constant through time, partly transmitted to the progeny;
- *E_Fi_* , an individual-level random term, constant through time, not transmitted;
- *E_Vit_* , an individual-level random term drawn annually.

We expect different impacts of the three individual-level random terms on population-level growth predictions, in particular on the population means and variances of tree diameter and annual tree diameter increment.

For instance, in the absence of competition, we expect that constant individual variation in vigor, both *G_I_* and *E_FI_*, increases the variance of tree diameter because of cumulative impacts of diameter increments through years, while annual fluctuation of individual performance, i.e. *E_Vi_*, has no such effect.

When competition occurs without mortality, we expect on the one hand that constant individual variation in vigor inflate the asymmetry of competition between neighbors, leading to an increase of the inter-individual coefficient of variation and, on the other hand, that competition globally decrease mean growth, resulting in a balanced effect on the mean and variance of tree diameter. When selective mortality by competition occurs, the consequences of constant individual variation in vigor become hardly predictable because competition intensity tends to increase the relative divergence between trees but the elimination of the weakest trees tends to homogenize the population and increase mean growth.

# Simulation plan

To better understand the multiple consequences of inter-individual variation in vigor on growth predictions, we simulated two stands of 2500 trees: one stand without competition (25 trees/ha) and one stand with competition (2500 trees/ha) where selective mortality started at age 43. For each stand, we simulated six different patterns of inter-individual variation in vigor characterized by the environmental and genetic variance terms as follows, with the same total phenotypic variance in all cases:

1. *VE_v_* = *VE_f_* = *VG* = 0: no inter-individual variation, i.e. the original deterministic growth model;
2. ***VE_v_* > 0**; *VE_f_* = *VG* = 0: only variable environmental effect, no constant individual effect;
3. ***VE_v_* > 0**; ***VE_f_* > 0**; *VG* = 0: environmental effect, 20% variable and 80% fixed;
4. *VE_v_* = 0; ***VE_f_* > 0**; *VG* = 0: only constant effect, purely environmental;
5. ***VE_v_* > 0**; ***VE_f_* > 0**; ***VG* > 0**: environmental effect, 20% variable and 80% fixed, and genetic effect;
6. *VE_v_* = 0; ***VE_f_* > 0**; ***VG* > 0**: only constant effects, environmental and genetic.

First, we examined the evolution of individual diameters and individual diameter increments during 100 years within a generation. Within a generation, the variation patterns (4) and (6) are strictly equivalent because they only have constant individual effects, while (3) and (5) are slightly different because the total environmental variance is lower in (5). Then, we simulated a regeneration phase and examined the values at the end of the second (non-overlapping) generation.

Note: the simulations below only represent one run of each case, replicated runs may result in slightly different mean and variance values without changing the main tendencies described here.

# 1. Without competition, within-generation


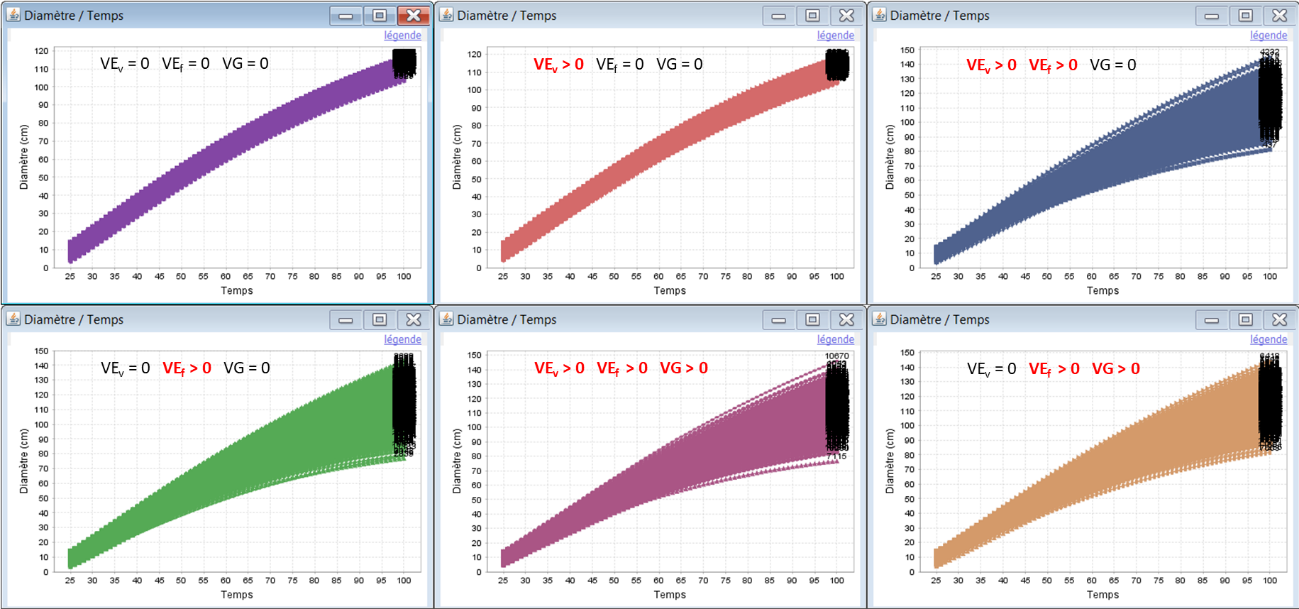


*Figure 1a: within-generation evolution of individual tree diameters (D) without competition. the black marks are the individual tree identifiers.*


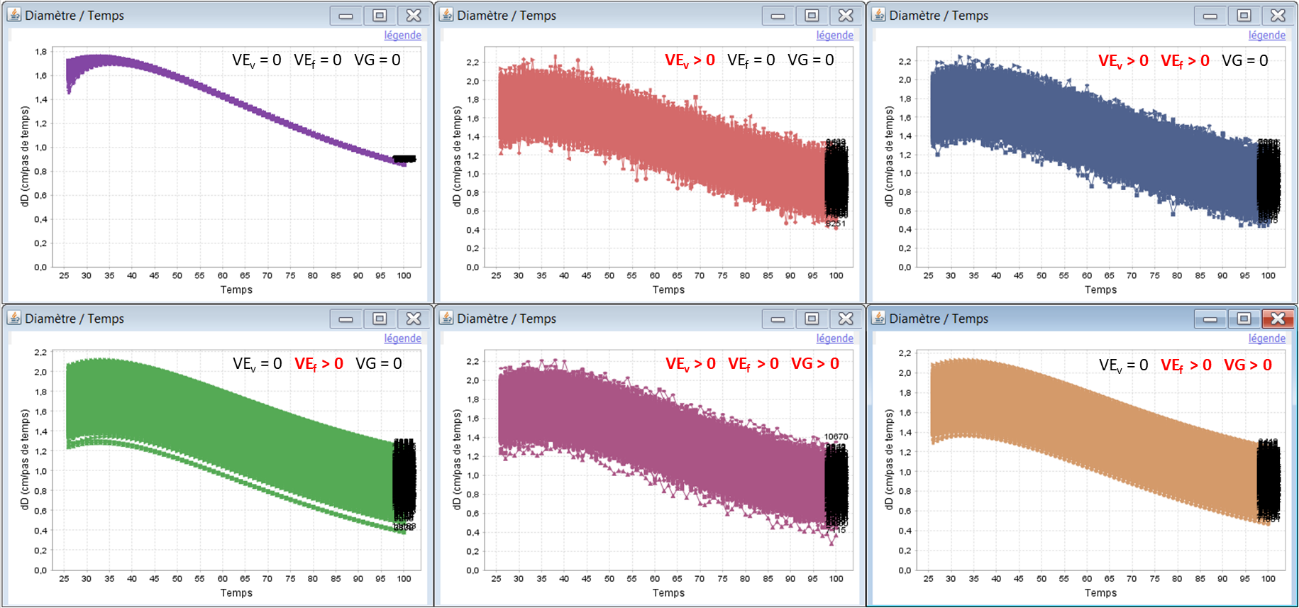


*Figure 1b: within-generation evolution of individual tree diameter increments (dD) without competition. The black marks are the individual tree identifiers.*

*Table 1: means and variances of individual tree diameters (D) and annual tree diameter increments (dD) at age 100 without competition.*

| ***VE_v_*** | ***VE_f_*** | ***VG*** | ***Mean(D)*** | ***Var(D)*** | ***Mean(dD)*** | ***Var(dD)*** |
| --- | --- | --- | --- | --- | --- | --- |
| 0 | 0 | 0 | 112 | 5 | 0.86 | 0 |
| 0.014 | 0 | 0 | 112 | 6 | 0.86 | 0.014 |
| 0.0028 | 0.0112 | 0 | 112 | 76 | 0.86 | 0.015 |
| 0 | 0.014 | 0 | 112 | 97 | 0.86 | 0.017 |
| 0.00196 | 0.00784 | 0.0042 | 112 | 78 | 0.86 | 0.015 |
| 0 | 0.0098 | 0.0042 | 112 | 90 | 0.86 | 0.016 |

- In the absence of competition, adding inter-individual variation in vigor in the model neither changes the predicted population mean diameter nor mean diameter increment.
- In the baseline deterministic growth model, the variance of annual increment becomes negligible at age 100 and the variance of tree diameter is only due to the initial stand variance before the simulation start.
- The variable environmental effects generate a variance of annual diameter increment that does not increase the population variance of tree diameter.
- Only the constant individual effects, fixed environmental and genetic effects, have an impact on the population variance of tree diameter.

# 2. With competition, within generation


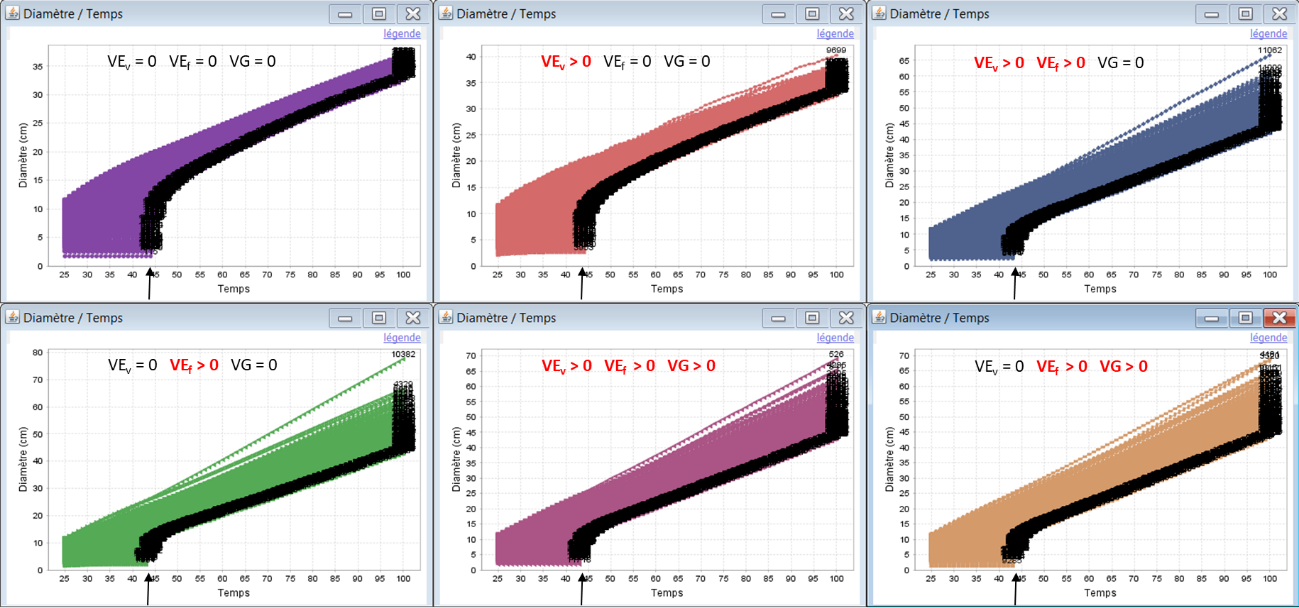


*Figure 2a: within-generation evolution of individual tree diameters (D) with competition. The arrows indicate the start of selective mortality; the black marks are the individual tree identifiers.*


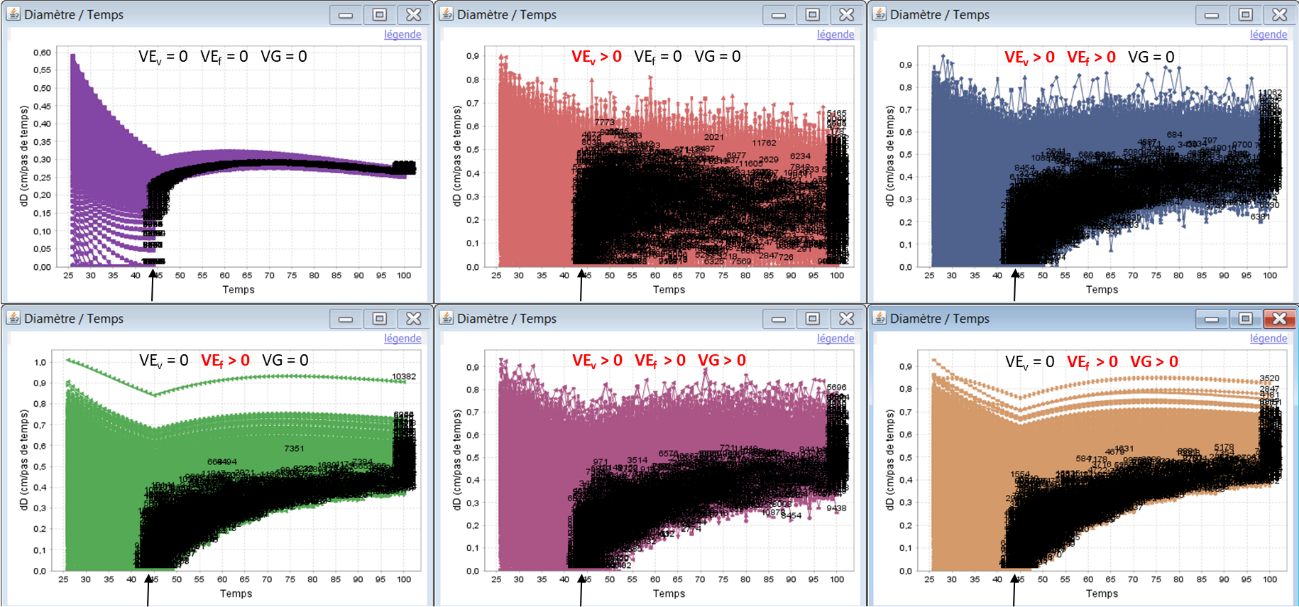


*Figure 2b: within-generation evolution of individual tree diameter increments (dD) with competition. The arrows indicate the start of selective mortality; the black marks are the individual tree identifiers.*

*Table 2a: means and variances of individual tree diameters (D) and annual tree diameter increments (dD) at age 43 with competition before mortality occurs)*

| ***VE_v_*** | ***VE_f_*** | ***VG*** | ***Mean(D)*** | ***Var(D)*** | ***Mean(dD)*** | ***Var(dD)*** |
| --- | --- | --- | --- | --- | --- | --- |
| 0 | 0 | 0 | 14 | 9 | 0.27 | 0.003 |
| 0.014 | 0 | 0 | 14 | 9 | 0.27 | 0.016 |
| 0.0028 | 0.0112 | 0 | 14 | 14 | 0.27 | 0.022 |
| 0 | 0.014 | 0 | 14 | 15 | 0.27 | 0.022 |
| 0.00196 | 0.00784 | 0.0042 | 14 | 14 | 0.27 | 0.022 |
| 0 | 0.0098 | 0.0042 | 14 | 14 | 0.27 | 0.022 |

Before selective mortality (self-thinning) occurs, at age 43:

- With competition before self-thinning, adding inter-individual variation in vigor in the model neither changes the predicted population mean diameter nor mean diameter increment.
- In the baseline deterministic growth model, the asymmetric interference between dominant and suppressed trees generates a variance of annual diameter increment (here, *Var(dD)*=0.003 at age 43), which progressively increases the variance of tree diameter.
- The variable environmental effects generate a variance of annual diameter increment that comes in addition to the competition-induced variation but does not inflate the population variance of tree diameter.
- There is a synergic effect between vigor and competitive advantage, which inflates the impact of constant individual effects on the variance of tree diameter and annual diameter increment.

*Table 2b: means and variances of individual tree diameters (D) and annual tree diameter increments (dD) at age 100 with competition after selective mortality*

| ***VE_v_*** | ***VE_f_*** | ***VG*** | ***Mean(D)*** | ***Var(D)*** | ***Mean(dD)*** | ***Var(dD)*** |
| --- | --- | --- | --- | --- | --- | --- |
| 0 | 0 | 0 | 34 | 1 | 0.26 | 0 |
| 0.014 | 0 | 0 | 35 | 2 | 0.26 | 0.014 |
| 0.0028 | 0.0112 | 0 | 47 | 18 | 0.48 | 0.007 |
| 0 | 0.014 | 0 | 49 | 26 | 0.50 | 0.006 |
| 0.00196 | 0.00784 | 0.0042 | 49 | 23 | 0.50 | 0.008 |
| 0 | 0.0098 | 0.0042 | 49 | 24 | 0.51 | 0.006 |

After selective mortality (self-thinning) has occurred, at age 100:

- Once selective mortality occurs, adding constant individual effects on vigor in the model increases both the predicted population mean diameter and mean diameter increment, while adding variable environmental effects has no such impact.
- In the baseline deterministic growth model, selective mortality progressively removes the suppressed trees, which reduces the competition-induced variation in annual diameter increment among surviving trees (here, *Var(dD)*=0) and reduces the variance of diameter.
- The variable environmental effects are not impacted by selective mortality and thus maintain a variance of annual diameter increment, with almost no impact on the variance of diameter.
- By contrast, the constant individual effects are impacted by selective mortality and cannot maintain the variance of annual diameter increment, but still they increase the variance of tree diameter because mean diameter is higher and asymmetric competition more intense than in the baseline.

# 3. Across generations

*Table 3: means and variances of individual tree diameters (D) and annual tree diameter increments (dD) at the end of the 1^st^ and the 2^nd^ generation (age 100), with competition and selective mortality, in the baseline growth model with no individual variation and the two models with the same genetic and environmental variances but different proportions of constant environmental variance.*

| ***VE_v_*** | ***VE_f_*** | ***VG*** | ***Mean(D)*** | ***Var(D)*** | ***Mean(dD)*** | ***Var(dD)*** |
| --- | --- | --- | --- | --- | --- | --- |
| *No individual variation, age 100* | | |  |  |  |  |
| 0 | 0 | 0 | 34 | 1 | 0.26 | 0 |
| *1st generation, age 100* | | |  |  |  |  |
| 0.00196 | 0.00784 | 0.0042 | 49 | 23 | 0.50 | 0.008 |
| 0 | 0.0098 | 0.0042 | 49 | 24 | 0.51 | 0.006 |
| *2^nd^ generation, age 100* | | |  |  |  |  |
| 0.00196 | 0.00784 | 0.0042 | 54 | 22 | 0.58 | 0.007 |
| 0 | 0.0098 | 0.0042 | 56 | 24 | 0.60 | 0.005 |

- For a given level of heritability (here, *h²*=0.3), the mean annual diameter increment and the mean diameter at the end of the 2^nd^ generation are higher with 100% constant environmental variance than 80% (*Mean(dD)*=0.60 vs 0.58, and *Mean(D)*=56 vs 54, respectively), which reveals a higher evolutionary rate of growth traits with 100% constant environmental variance than 80%.
- This can be explained considering the slightly higher selection differential on vigor at the end of the first generation with 100% constant environmental variance than 80% (*Mean(dD)*=0.51 vs 0.50); this higher selection differential relates to higher competition intensity and higher contribution of the constant individual effects on the phenotype. As a consequence of higher selection differential on vigor, trees in the next generation have an increased growth, which intensifies selective mortality by competition during the 2^nd^ generation.
- The slight difference in the variance of annual diameter increment at the end of each generation (*Var(dD)*) between 100% constant environmental variance and 80% is simply the contribution of the variable environmental effects (*VE_v_*).
