# Supplementary material for: A demo‐genetic model shows how silviculture reduces natural density‐dependent selection in tree populations
Source: Evol Appl. 2023 Oct 13;16(11):1830–44. doi: 10.1111/eva.13606 (PMC10681482; doi:10.1111/eva.13606)
Supplement: Supplementary file 5 — Appendix S5 [file EVA-16-1830-s003.docx]

Appendix 5 - Supplementary Figures


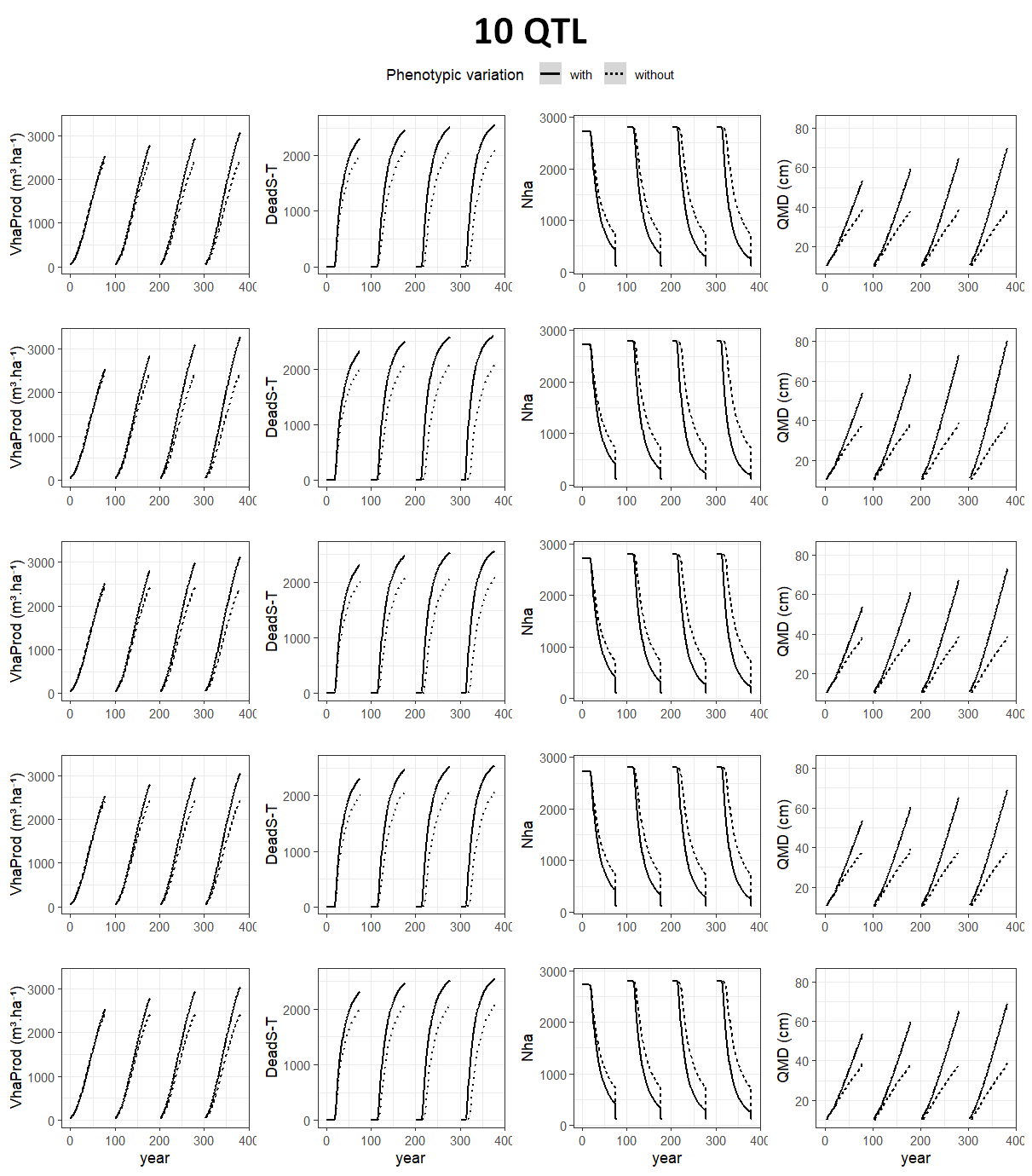


**Figure S3 (Part 1).** Dynamics of forest productivity, demography and tree size in the unthinned long cycle scenario (U-long) without disturbance, with or without phenotypic variation (line types): total timber volume produced per hectare (*VhaProd*), number of deaths by self-thinning per hectare (*DeadS-T*), number of trees per hectare (*Nha*) and quadratic mean diameter (QMD). Part 1 illustrates the five genetic setups of 10 QTL (*in rows*); see part 2 for 50 QTL. The 95% intervals over 10 replicates, represented by shaded areas, are imperceptible. Trees are 25-years old when simulations start at year 0. The pre-recruitment period is deliberately not represented because the phenotype is not considered in the model during this phase.


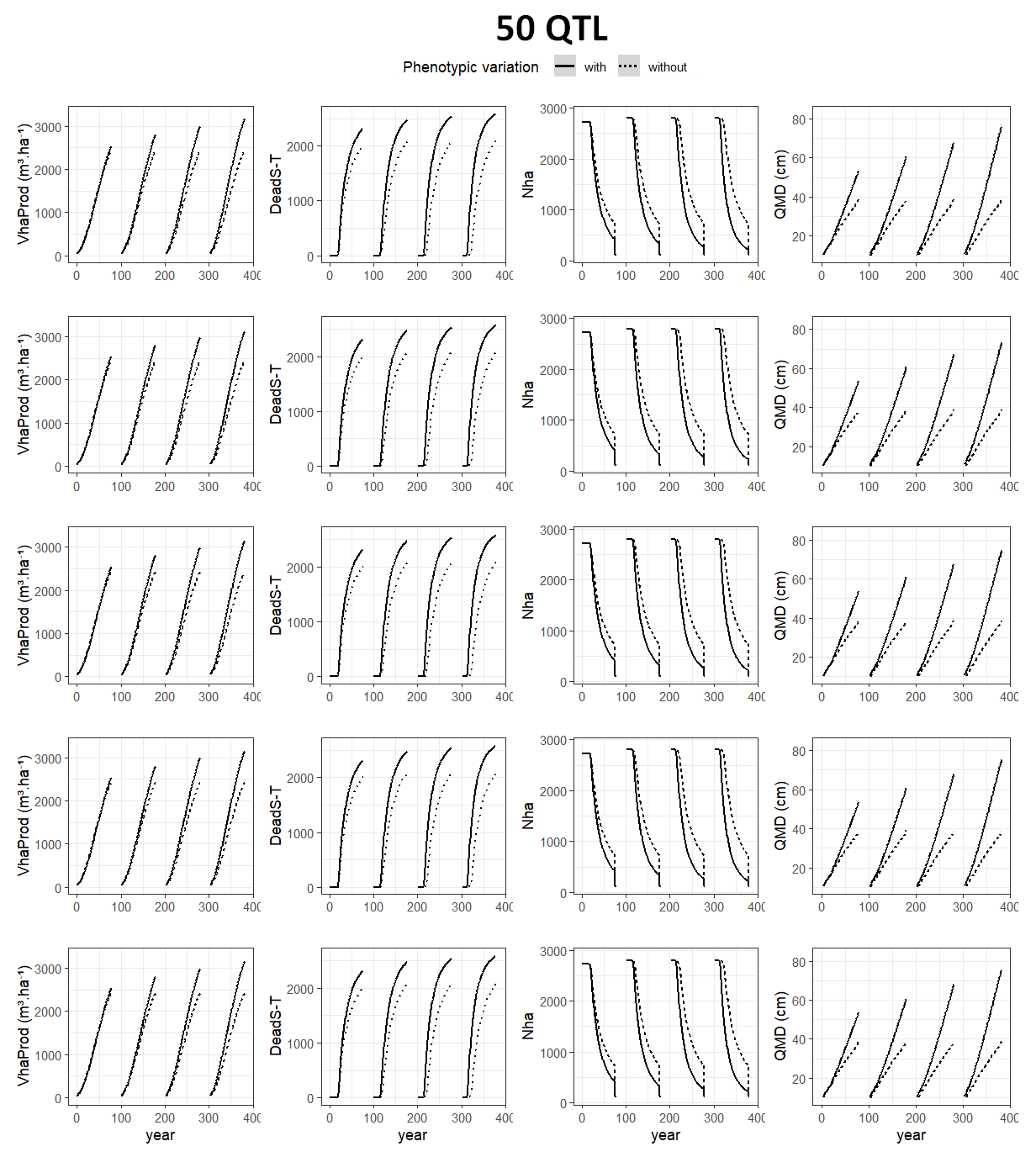


**Figure S3 (Part 2).** Dynamics of forest productivity, demography and tree size in the unthinned long cycle scenario (U-long) without disturbance, with or without phenotypic variation (line types): total timber volume produced per hectare (*VhaProd*), number of deaths by self-thinning per hectare (*DeadS-T*), number of trees per hectare (*Nha*) and quadratic mean diameter (QMD). Part 2 illustrates the five genetic setups of 50 QTL (*in rows*); see part 1 for 10 QTL. The 95% intervals over 10 replicates, represented by shaded areas, are imperceptible. Trees are 25-years old when simulations start at year 0. The pre-recruitment period is deliberately not represented because the phenotype is not considered in the model during this phase.

**
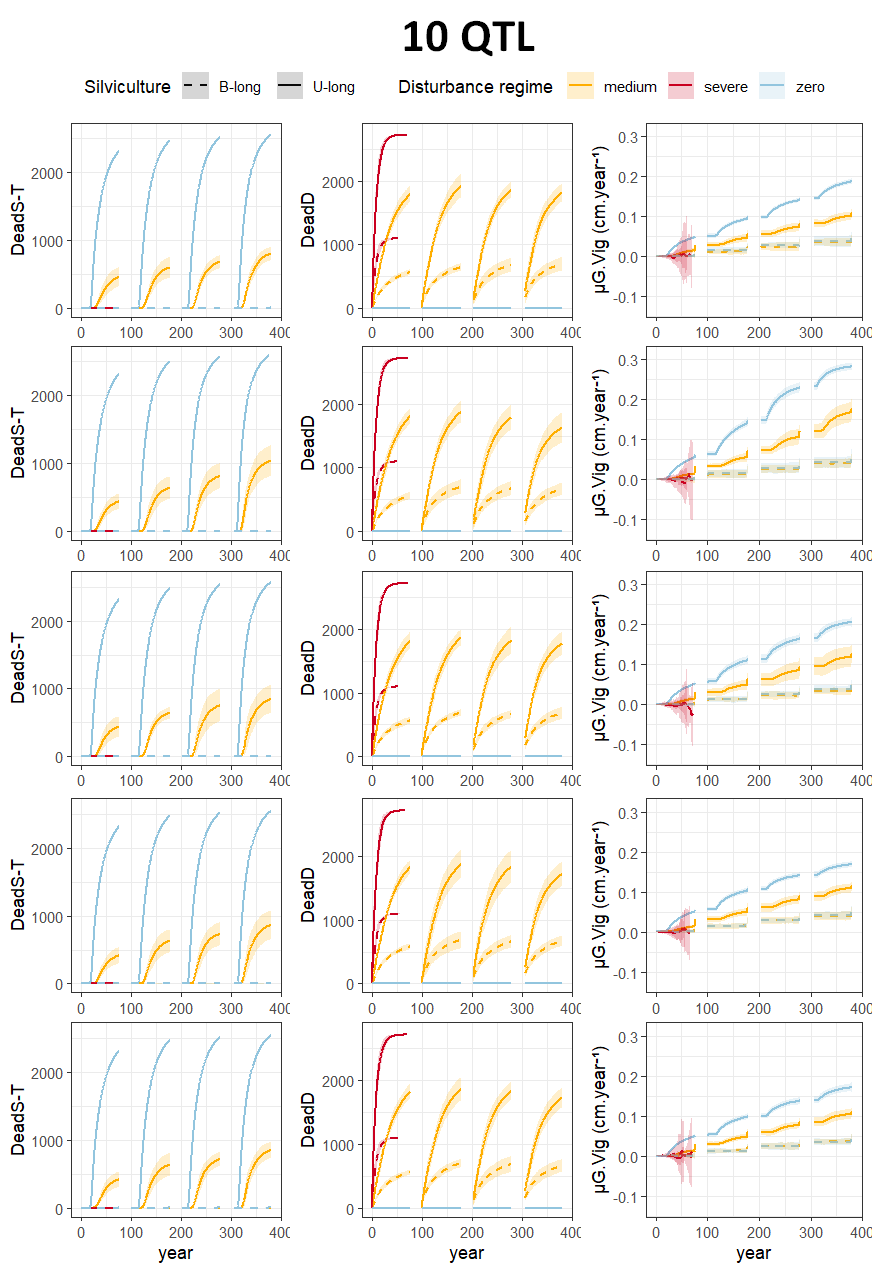
**

**Figure S4 (Part 1).** Dynamics of mortality and genetic changes in the long cycle scenarios with phenotypic variation, unthinned (*U-long*) or with baseline thinning (*B-long*) and three disturbance regimes (in colors): deaths by self-thinning (*DeadS-T*), deaths by disturbance (*DeadD*) and population genetic mean of vigor (*µG.Vig*). Part 1 illustrates the five genetic setups of 10 QTL (*in rows*); see part 2 for 50 QTL. The shaded areas represent the 95% intervals over 10 replicates for each scenario. With the severe disturbance regime, the population collapsed during the first cycle. Trees are 25-years old when simulations start at year 0. The pre-recruitment period is deliberately not represented because the phenotype is not considered in the model during this phase.

**
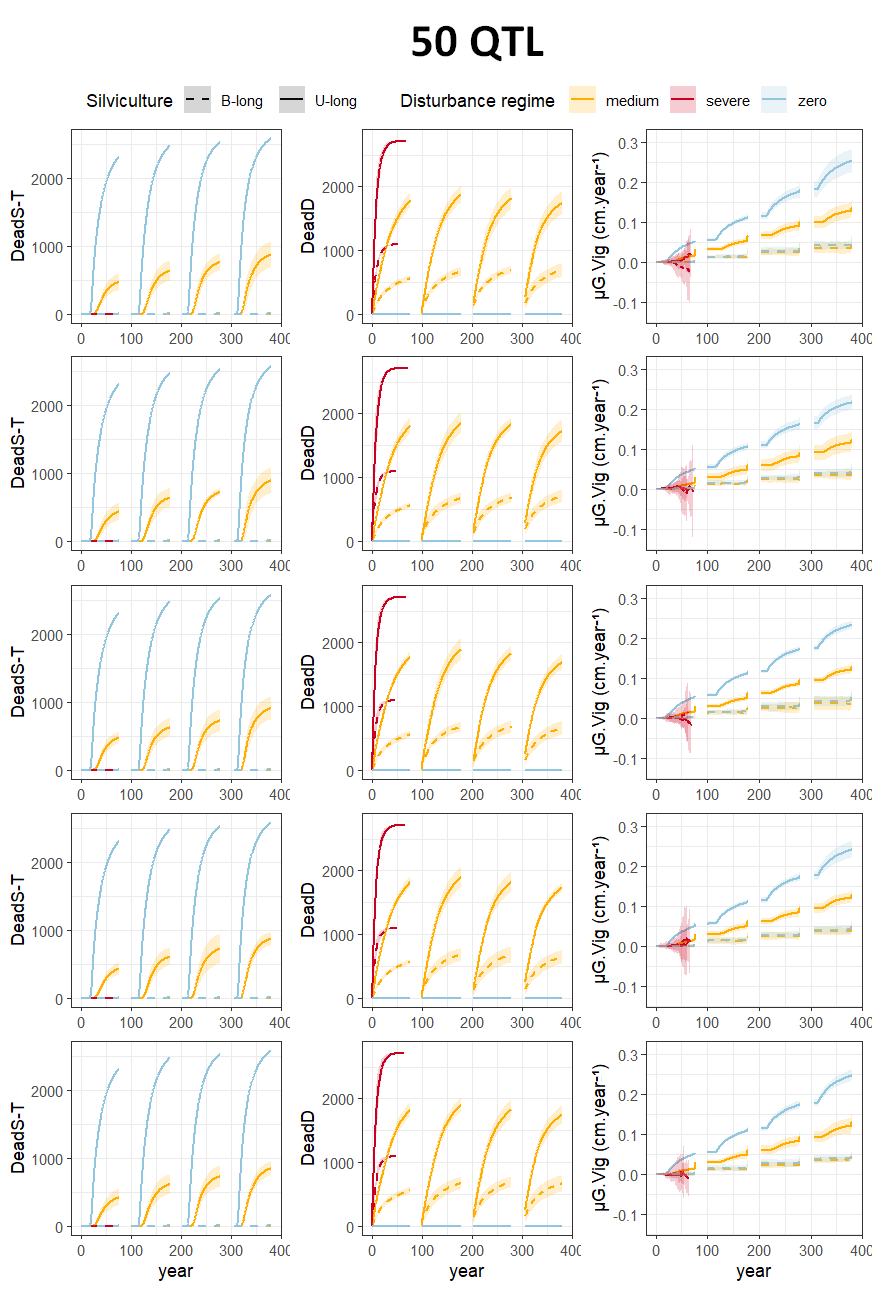
**

**Figure S4 (Part 2).** Dynamics of mortality and genetic changes in the long cycle scenarios with phenotypic variation, unthinned (*U-long*) or with baseline thinning (*B-long*) and three disturbance regimes (in colors): deaths by self-thinning (*DeadS-T*), deaths by disturbance (*DeadD*) and population genetic mean of vigor (*µG.Vig*). Part 2 illustrates the five genetic setups of 50 QTL (*in rows*); see part 1 for 10 QTL. The shaded areas represent the 95% intervals over 10 replicates for each scenario. With the severe disturbance regime, the population collapsed during the first cycle. Trees are 25-years old when simulations start at year 0. The pre-recruitment period is deliberately not represented because the phenotype is not considered in the model during this phase.


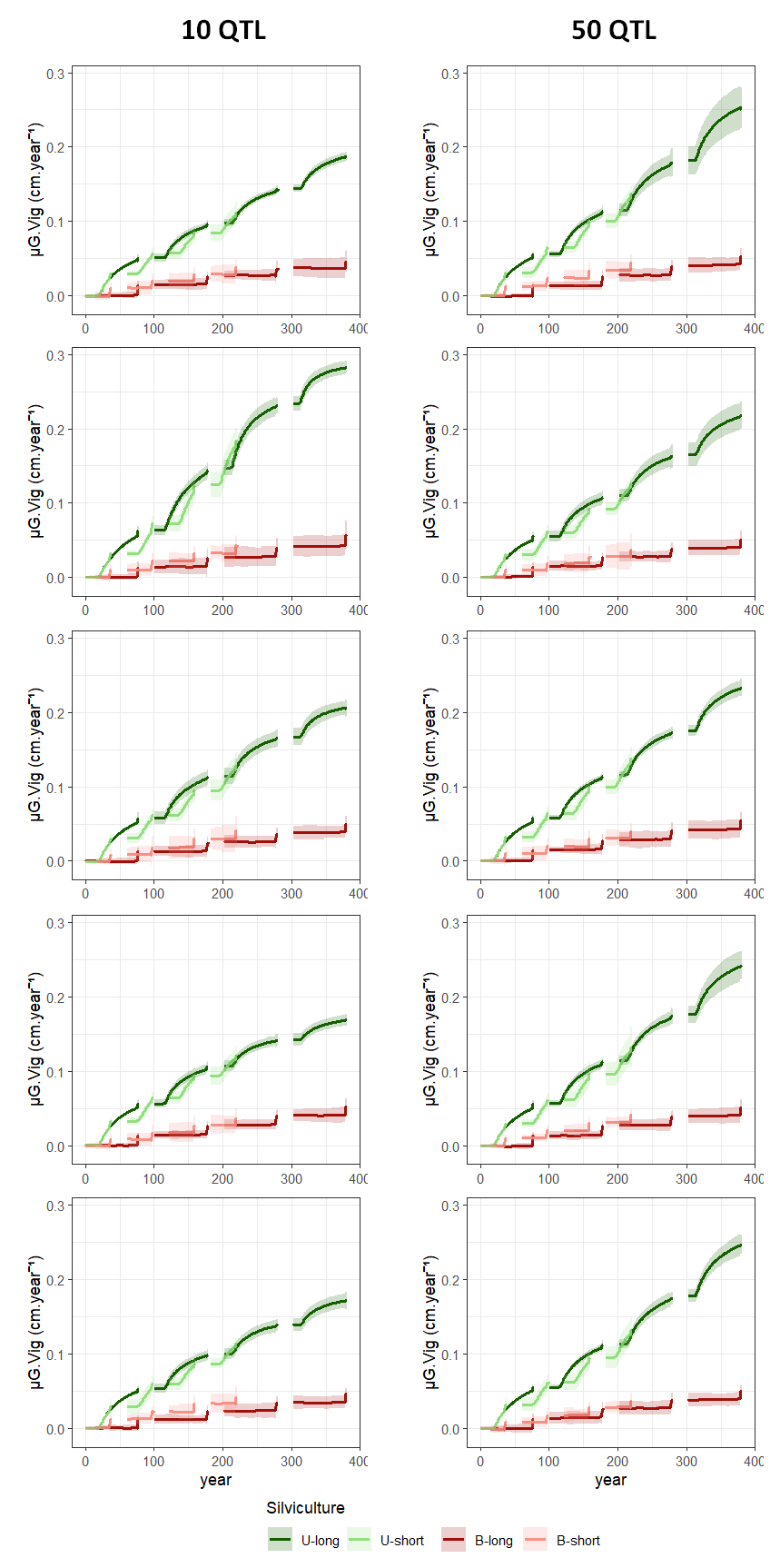


**Figure S5.** Dynamics of the population genetic mean of vigor (*µG.Vig*) in four scenarios, long or short cycle unthinned (*U-long*, *U-short*, respectively, in colors) or with baseline thinning (*B-long*, *B-short*, respectively). This figure illustrates the genetic setups for 10 QTL (*n*= 5), on the left, and 50 QTL (*n=* 5), on the right. The shaded areas represent the 95% intervals over 10 replicates for each scenario. Trees are 25-years old when simulations start at year 0. The pre-recruitment period is deliberately not represented because the phenotype is not considered in the model during this phase.


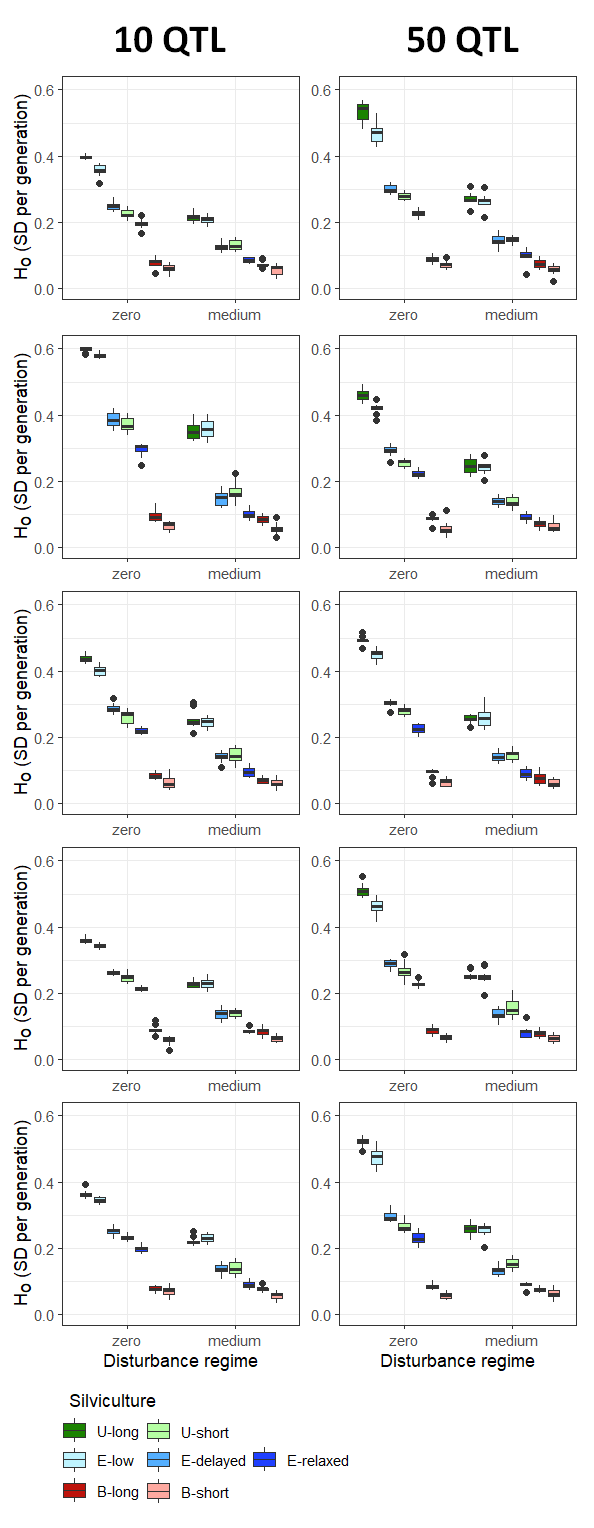


**Figure S6.** Average evolutionary rate (*H_0_*) in vigor after four generations for the different silvicultural scenarios (in colors) and disturbance regimes (x-axis, the severe disturbance regime is not represented because it led to population collapse). The boxplots illustrate the distribution of values over 10 replicates in each case. This figure illustrates the genetic setups for 10 QTL (*n*= 5), on the left, and 50 QTL (*n*= 5), on the right.

*Silvicultural scenarios: unthinned long cycle (U-long); unthinned short cycle (U-short); baseline thinning long cycle (B-long); baseline thinning short cycle (B-short); exploratory low intensity thinning long cycle (E-low); exploratory delayed thinning long cycle (E-delayed); exploratory relaxed thinning long cycle (E-relaxed).*
